# Competing methods of divergence time estimation recover diverse evolutionary timescales

**DOI:** 10.1098/rspb.2026.0635

**Published:** 2026-10-07

**Authors:** Sidney E. L. Davies, Alessia Tasca, Sandra Álvarez-Carretero, Christopher Brochu, Davide Pisani, Philip C. J. Donoghue

**Affiliations:** ^1^ Bristol Palaeobiology Group, School of Earth Sciences, University of Bristol, Bristol BS8 1TQ, UK; ^2^ Bristol Palaeobiology Group, School of Biological Sciences, University of Bristol, Bristol BS8 1TQ, UK; ^3^ Department of Biotechnology and Biosciences, University of Milano-Bicocca, Milan, 20126, Italy; ^4^ Department of Genetics, Evolution and Environment, University College London, London WC1E 6BT, UK; ^5^ School of Earth, Environment and Sustainability, University of Iowa, IA, 52242, USA

**Keywords:** molecular clock, calibration, crocodiles, fossilized birth–death (FBD), fossil record, MrBayes

## Abstract

Molecular clocks convert evolutionary distances into geological times to infer time-scaled phylogenies. Methods differ in terms of fossil calibration strategy (node versus tip) and whether they use molecular and/or morphological data, but their relative performance remains unclear. Given their well preserved fossil record, we use Crocodylia to interrogate common approaches to timescale estimation using a combination of calibration strategies and datatypes in MrBayes. We found that calibration strategies had a much greater impact than datatype, with node-calibrated timescales producing younger ages than tip calibration. The older ages recovered by tip-calibrated approaches imply an Early Cretaceous origin (approx. 125–102 Ma) of Crocodylia, inconsistent with the biostratigraphic record, while node calibration produces more credible estimates, including a Late Cretaceous origin (approx. 95–78 Ma). Performing tip calibration with a fossilized birth–death process produced an intermediate timescale, estimating an Early–Late Cretaceous origin (approx. 118–95 Ma). These timescales support materially distinct scenarios for crocodylian evolution. We show that these disparities among calibration strategies are driven by differences in the structure of their underlying priors, with tip-calibrated timescales biased toward unrealistically ancient ages. Our results highlight the need for future analyses to explore hybrid calibration strategies that treat node and tip calibration as complementary, rather than competing, approaches.

## 1. Introduction

The fossil record is an invaluable but imperfect temporal archive of evolutionary history that has led palaeontologists and evolutionary biologists alike to seek alternative methods for calibrating evolutionary events to geological time [1]. The most widely adopted method is the molecular clock, which combines observed genetic distances between species with calibration densities derived from the fossil record to estimate rates of molecular evolution and infer divergence times [2,3]. In particular, timetree inference is now dominated by Bayesian relaxed-clock models that explicitly model among-lineage rate variation and integrate over-uncertainties in the structure of the palaeontological record [4,5]. Such methods include node calibration, where the timing of specific lineage divergences is constrained by user-specified probability distributions based on interpretations of the fossil record, or tip calibration, where divergence times are extrapolated from both the phylogenetic position and stratigraphic ranges of extinct taxa [6–10]. As a result, a complex suite of methods is currently available for incorporating distinct calibration strategies for molecular, morphological or combined datasets, facilitating the analysis of living and/or fossil species, conditioned on fixed topologies or co-estimating a tree topology and species divergence times [11–13].

Does this diversity of methods estimate evolutionary time equally well? Node calibrations are usually based on qualitative assessments of the palaeontological record and are limited to using the oldest credible fossil occurrences of clades [14]. Interactions among calibrations and the tree prior can cause ‘effective’ calibrations (marginal densities) to deviate from user-specified node-age constraints (calibration densities) [15,16]. Tip calibration overcomes this issue by integrating fossils directly into a phylogeny alongside their living relatives, although this method has been known to produce implausibly ancient estimates [17,18]. More recently, the fossilized birth–death (FBD) process, which combines parameters for speciation, extinction and fossil sampling under a unified phylodynamic prior, has additionally been proposed as a solution for avoiding subjective interpretations of the fossil record while retaining an explicit model-based approach [19–23]. In this study, we assess relative performance among dating methods using crocodylians, a group with a well resolved phylogeny of living species and a well sampled, intensively studied fossil record [24].

## 2. Material and methods

### (a) Analyses

To evaluate performance, we compared marginal (effective) priors and posterior time densities across node- and tip-calibrated analyses using MrBayes v. 3.2.7a [25]. For node calibration, the effective prior can be explicitly derived by running the analysis without sequence or trait data. For tip-calibrated analyses involving co-estimation, marginal calibration densities were approximated by sampling node ages from the prior conditioned on a fixed extant topology, with the position of fossil taxa informed solely by their ages (without character data). This resulted in three analyses when sampling from the prior under three distinct calibration strategies: node calibration, tip calibration under the uniform tree prior, and tip calibration under the FBD process (see §2d). For posterior timescales, additional analyses were performed corresponding to different datatypes, resulting in six overall: three node-calibrated variants (one molecular, one morphological, one combined-evidence) and three tip-calibrated variants (one total-evidence, one morphological and one total-evidence with an FBD tree prior). It is worth stressing that ‘combined-evidence’ refers to the joint analysis of molecular and morphological characters, whereas ‘total-evidence’ refers to the joint analysis of molecular and morphological characters with serially sampled fossil tips. All analyses were conditioned on a fixed extant backbone topology inferred using IQ-TREE 2 [26]; however, tip-calibrated approaches co-estimated the phylogenetic placement of fossil taxa with divergence times.

### (b) Phylogenetic dataset

Our molecular dataset consists of a 9284-nucleotide alignment for 24 living crocodylian taxa, representing all species for which there are molecular data available. The molecular matrix comprises 10 nuclear genes and three mitochondrial loci, used previously in [27]. A best-fit partitioning scheme for molecular data was established based on a previous analysis using PartitionFinder, with five alignment blocks assigned an independent substitution model [27,28] (see supplementary material). For analyses involving morphology, a phenotypic dataset comprising 330 discrete characters was used, also from previously published data [29]. This encompasses character data for 24 living taxa, as well as for 61 additional fossil species for analyses involving extinct taxa. Metadata for fossil taxa, including specimen ID and literature source, are available in [29, appendix 1]. The morphological dataset was analysed under the Mk+Γ model [30], and became the sixth alignment block when analysed jointly with molecular data.

### (c) Calibrations

Six node calibrations were specified following best practice [31]. As an example, we specified a minimum age for the root based on the oldest unequivocal in-group fossil taxon *Leidyosuchus canadensis* (holotype: AMNH 5342 [32]), as supported by a recent comprehensive crocodylian phylogeny incorporating fossil species [27]. The root age was set to a minimum of 74.99 Ma based on U–Pb radiometric dating of the Dinosaur Park Formation, where all specimens of *L. canadensis* have been discovered [33]. An upper bound of 95 Ma is characterized by the absence of crown-group fossils compatible with the presence of morphologically comparable eusuchians such as *Portugalosuchus azenhae*, indicating that the root diversification of crown crocodylians postdates this time interval [34,35].

To appropriately account for the uncertainty of node constraints, hard maxima were applied only when the fossil record provided strong evidence that older crown occurrences are improbable [36]. For example, we did not apply a hard maximum for crown Caimaninae because several older taxa have been repeatedly recovered in unstable positions in phylogenetic analyses, which could plausibly represent crown caimans [37]. Since MrBayes does not permit soft bounds, we followed the approach described in [14] and specified offset-exponential distributions with a lower bound (*t*_L_) and rate parameter (*θ*) for minimum-age constraints, where the mean is *θ*^−1^ + *tL*. Under this implementation, larger values of *θ* mean that the true age of divergence is likely to be close to *tL* (similar to ‘hard’ bounds). In this study, we used *θ* = 1/*tL* so that the mean of our distributions was ${\left(\frac{1}{tL}\right)}^{-1}+tL$ = 2*tL*. While other plausible implementations are possible, we chose the aforementioned to account for the non-negligible probability that the true age of divergence is significantly older than the fossil minimum. By using relatively uninformative calibrations, we avoid forcing analyses toward prior distributions pre-emptively supporting specific age ranges. While alternative methods exist for analytically deriving node calibrations from the fossil record [38–41], assessing our marginal prior densities in light of the underlying palaeontological evidence indicated that our offset-exponential calibrations were sufficiently permissive to accommodate multiple scenarios of evolution, while still penalizing unrealistically older dates (see Discussion).

For tip-calibrated analyses, fossil tips were assigned age ranges corresponding to the oldest and youngest stratigraphic occurrences, as implemented in previous total-evidence analyses [20,22,42]. For singletons known from a single stratigraphically unique occurrence, age ranges were representative of the uncertainty related to the dating of this specific occurrence. We then sampled the age of each fossil from a uniform distribution—therefore, the species’ stratigraphic range is treated as uncertainty in the age of a single fossil. This approach avoids fixing fossil ages to point estimates, which can bias divergence-time inference [43]. Instead, age uncertainty is directly integrated into the dating analysis. In addition, a uniform calibration density for the root was set to a minimum of 86.3 Ma (corresponding to the first appearance of the oldest stem crocodylian) and a maximum of 125.5 Ma (corresponding to the last occurrence of the outgroup *Bernissartia fagesii*). This constraint was deliberately broader than its node-calibrated counterpart owing to the inclusion of fossil species, which shifts the root of the tip-calibrated phylogeny outside crown Crocodylia to the most recent common ancestor of Eusuchia. The full dating justifications for all node and tip calibrations are available in the supplementary material.

### (d) Priors

We specified the following settings in MrBayes for all analyses. To account for rate variation across lineages, we set an independent gamma-rate relaxed-clock model [44] and assigned a gamma prior (*α* = 2, *β* = 67.6) to the mean rate. Values of *α* and *β* were calculated using an in-house R script (see supplementary material). For node-calibrated analyses, the tree prior was conditioned on a birth–death process with species sampling (BDS), with hyperpriors for speciation *λ* ∼ Uniform(0, 10)), relative extinction *r* ∼ Beta(1, 1) and extant sampling probability *ρ* = 0.93. Since the BDS prior is mis-specified when applied to phylogenies with serially sampled tips, such as those including fossil taxa, tip-calibrated analyses were instead conducted using either a uniform tree prior or the FBD process. For the latter, the tree prior treated *ρ* as a fixed parameter, with hyperpriors placed on net diversification rate *d* = *λ*(1 – *r*) ∼ Exponential(10), relative extinction rate *r* ∼ Beta(1, 1)) and fossil sampling rate *ψ* ∼ Beta(1, 1). This allowed us to assess how probabilistically modelling heterochronous total-evidence datasets influences timetree inference in comparison with a vague tree prior. It is important to note that our FBD model used a constant-rate process with stratigraphic ranges representing uncertainty in the ages of individual fossils. More flexible skyline models, which allow the FBD model parameters to vary through time [45], as well as extensions that integrate over multiple fossil occurrences across a species’ stratigraphic range, may provide more appropriate frameworks for modelling fossil sampling rates [46].

### (e) Markov chain Monte Carlo

Each analysis was run under the Bayesian Markov chain Monte Carlo (MCMC) approach implemented in MrBayes. To explore the state space, we ran four Markov chains with a minimum of 10 000 000 iterations and a maximum of 75 000 000 iterations for more parameter-rich analyses such as those co-estimating time and topology. Results were verified for convergence using Tracer [47], while effective sample size (ESS) scores of more than 200 indicated good mixing across MCMC chains. To assess congruency between inferred timescales, we calculated three performance metrics for each analysis to compare (i) the relative uncertainty associated with node-age estimates (coefficient of variation, calculated as Highest Posterior Density (HPD) width/mid-value), (ii) the overall duration of the inferred evolutionary history (tree length) and (iii) the degree of agreement with the node-calibrated molecular analysis. The latter was used as a reference timescale for comparison, with concordance correlation coefficient (CCC) values quantifying concordance among analyses.

## 3. Results

### (a) Prior timescales

Performing analyses without sequence data produced three initial timescales for modern crocodiles conditioned on the effective prior alone (figure 1). For node calibration, this resulted in an origin of 93.7–74.9 Ma (95% highest prior density), which closely corresponds to the user-specified calibration density used to constrain the root age (95–74.9 Ma, uniform distribution) (figure 2). The same behaviour was observed with the single other uniform calibration (Alligatoridae, 81.19–62.53 Ma). For other calibrated nodes, we specified offset exponential priors, which resulted in much wider age intervals, but with a diminishing probability of divergence approaching the maxima (i.e. crown Caimaninae: 61.03–18.64 Ma and Crocodylidae: 70.06–21.18 Ma) (figure 2). For tip calibration, there are no calibration densities to constrain node ages: the marginal time densities emerge indirectly from several model components. Using a uniform tree prior, this resulted in older marginal time densities, including an origin of 124.66–112.54 Ma. Under the FBD process, an origin time of 111.72–86.06 Ma was inferred. The resulting effective prior for FBD displayed stronger structural heterogeneity, with older divergence times inferred for fossil-rich clades (i.e. Longirostres: 97.46–76.43 Ma) and younger node ages in clades lacking early fossil representatives in our dataset (i.e. *Caiman* split: 14.14-4.53 Ma). The summed branch lengths for prior timescales equalled 641 Myr (node calibration), 1 221 Myr (tip calibration— uniform) and 678 Myr (tip calibration—FBD). The corresponding coefficients of variation (CVs) were 1.05, 0.56 and 0.53.

**Figure 1.**
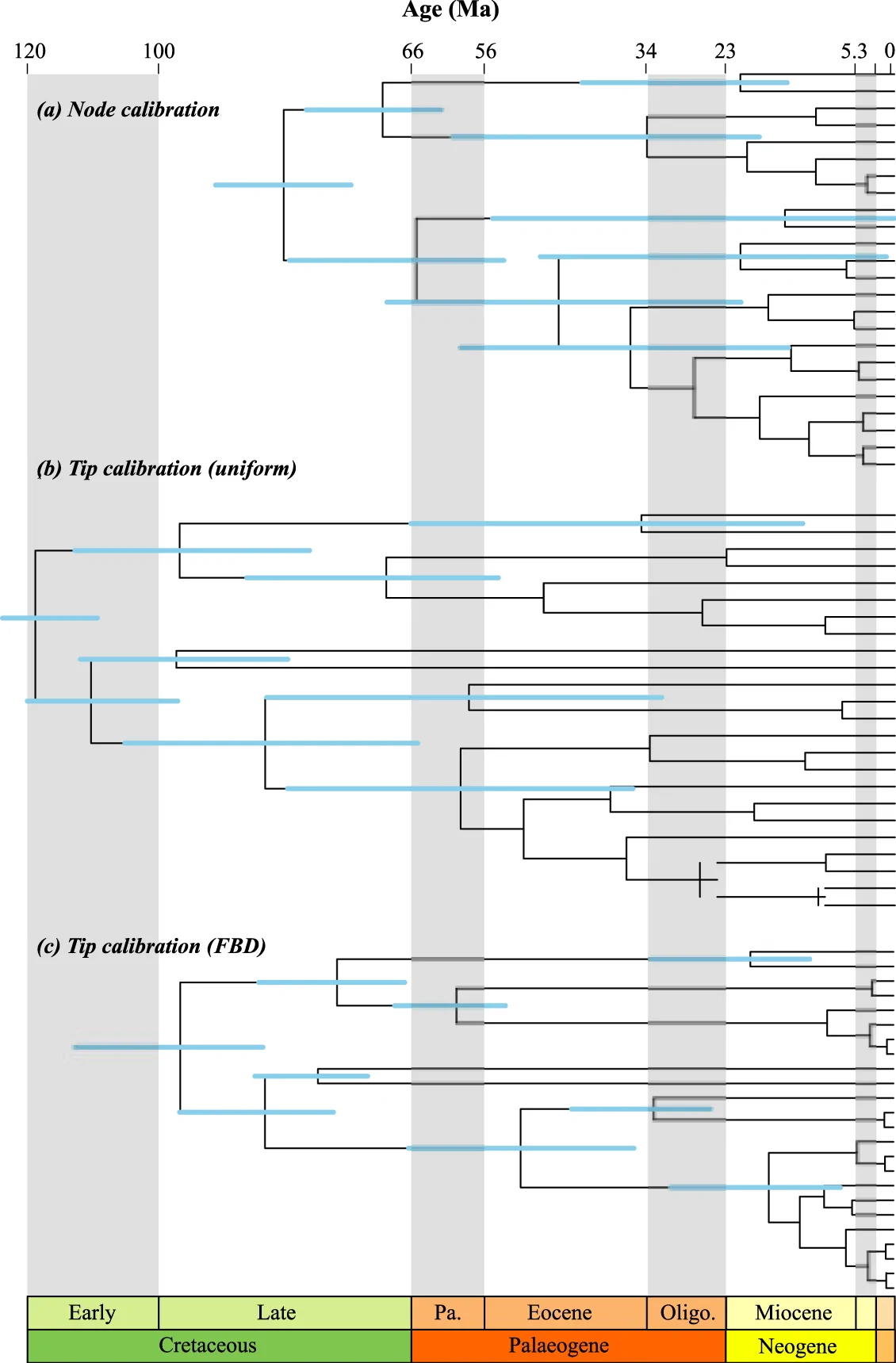
Effective prior timescales obtained from running the inference without character data for (*a*) node calibration, (*b*) tip calibration (uniform tree prior) and (*c*) tip calibration (fossilized birth–death (FBD)). Nodes represent mean ages with uncertainty widths (blue bars) indicating the Highest Posterior Density (HPD) for key clades. HPD widths are restricted to key clades for readability. For tip-calibrated timescales, fossil taxa have been pruned to provide a like-for-like comparison with node calibration. Pa., Palaeocene; Oligo., Oligocene.

**Figure 2.**
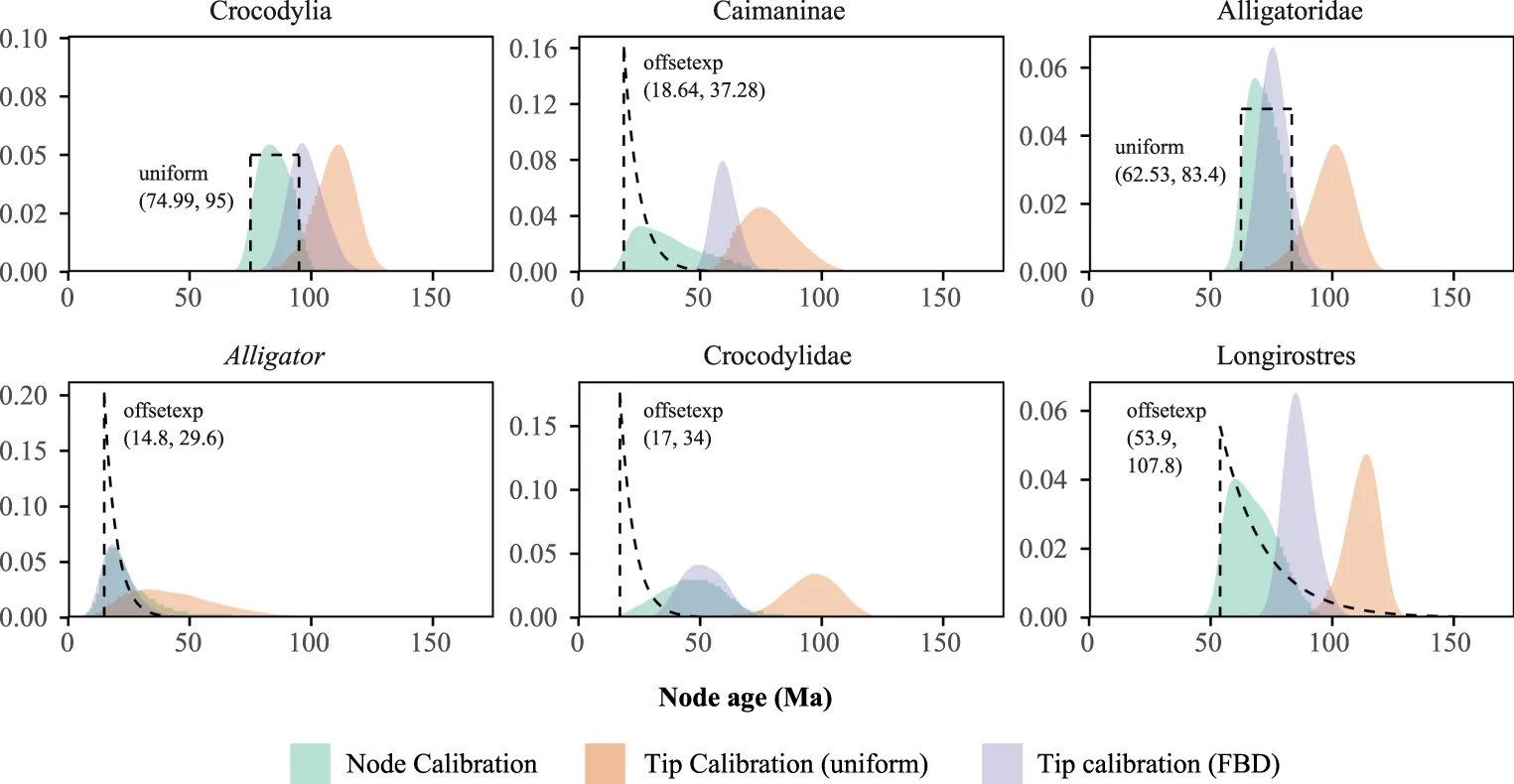
Comparison of effective prior distributions obtained from running the inference without character data for (*a*) node calibration, (*b*) tip calibration (uniform tree prior) and (*c*) tip calibration (fossilized birth–death (FBD)). Dashed lines represent user-specified node calibration prior densities summarizing our best understanding of the relevant palaeontological, stratigraphic and phylogenetic evidence. Adjacent text represents the explicit calibration densities implemented in MrBayes. offsetexp, offset-exponential distribution.

### (b) Node calibration

We inferred the best-scoring maximum-likelihood tree with IQ-TREE 2, producing an extant backbone topology consistent with recent investigations into crocodylian phylogenetics, including a putative sister relationship between *Gavialis gangeticus* and *Tomistoma schlegelii* (figure 3*a*). While fixing this topology, the inferred node-calibrated molecular timescale estimated the root divergence to 95–88 Ma (95% highest posterior density), during the Turonian stage of the Late Cretaceous and bounded by the user-specified crown calibration density. Except for Alligatoridae, whose estimated 95% HPD interval (68.4–62.5 Ma) spans the Cretaceous–Palaeogene (K–Pg) boundary, all other divergences were inferred to take place during the Cenozoic, including: Longirostres (62.7–54 Ma), Crocodylidae (35.2–20.6 Ma), crown Caimaninae (40–23.3 Ma), Gavialidae (30.9–14 Ma), crown Osteolaeminae (27–13.5 Ma) and *Alligator* (52.2–31.6 Ma). The most recent major clade to diverge was *Crocodylus*, which was estimated to have radiated during the Miocene epoch (20.3–10.8 Ma), spanning multiple stages. The total tree length and CV of the node-calibrated molecular timescale were 581 and 0.46 Myr, respectively.

**Figure 3.**
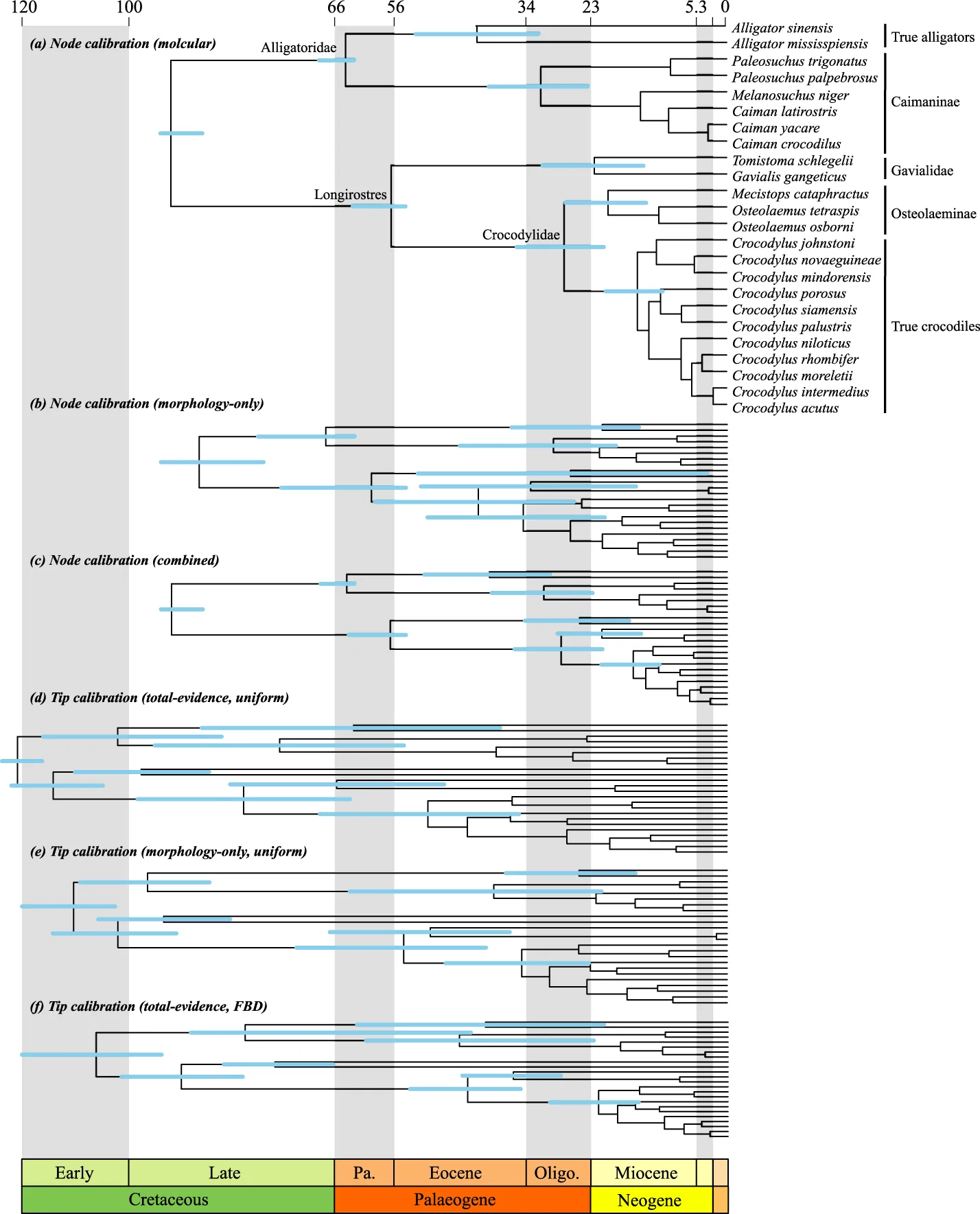
Posterior timescale for six dating analyses of crocodylian evolution for (*a*) node calibration (molecular), (*b*) node calibration (morphology), (*c*) node calibration (combined), (*d*) tip calibration (total-evidence with a uniform tree prior), (*e*) tip calibration (morphology with a uniform tree prior) and (*f*) tip calibration (total-evidence with fossilized birth–death (FBD)). Nodes represent mean ages, with uncertainty widths (blue bars) indicating the highest posterior density (HPD) for key clades. HPD widths are restricted to key clades for readability. For tip-calibrated timescales, fossil taxa have been pruned to provide a like-for-like comparison with node calibration. Pa., Palaeocene; Oligo., Oligocene.

Further node-calibrated analyses (morphology-only and combined molecular and morphology datasets) also recovered Late Cretaceous origins consistent with the root calibration density (95 Ma) (figure 3*b*,*c*). However, the lower bound inferred in the morphology-only analysis is closer to the K–Pg boundary, during the Campanian stage (77.8 Ma). CCC values comparing each timescale with the molecular node-calibrated analysis were 0.87 and 0.99 for the morphology-only and combined analyses, respectively (figure 4). In the combined analysis, all post-root divergences were inferred after the K–Pg boundary, except for Alligatoridae, whose upper bound slightly predates it (68.2–62.5 Ma). In the morphology-only analysis, the inferred upper bounds for both Alligatoridae and Longirostres predate the K–Pg boundary (78.7–62.5 and 74.8–54.0 Ma, respectively). Inferred divergence estimates for other major clades were: crown Caimaninae (morphology: 44.8–18.6 Ma, combined: 39.5–22.6 Ma), Gavialidae (51.6–3.4 Ma, 33.9–16.5 Ma), Crocodylidae (59.1–25.7 Ma, 35.7–20.9 Ma) and crown Osteolaeminae (51.4–15.3 Ma, 28.5–14.4 Ma). In the combined analysis, *Crocodylus* represented the youngest divergence, during the Miocene (21.08–11.36 Ma). Under the morphological clock, *Crocodylus* diverged earlier and *Alligator* later (36.17–14.8 and 50.29–20.54 Ma, respectively), with wide HPD intervals limiting resolution to specific geological stages. The morphology-only analysis had a high CV of 0.87 and a total tree length of 690 Myr; the corresponding values for the combined-data analysis were 0.46 and 584 Myr.

**Figure 4.**
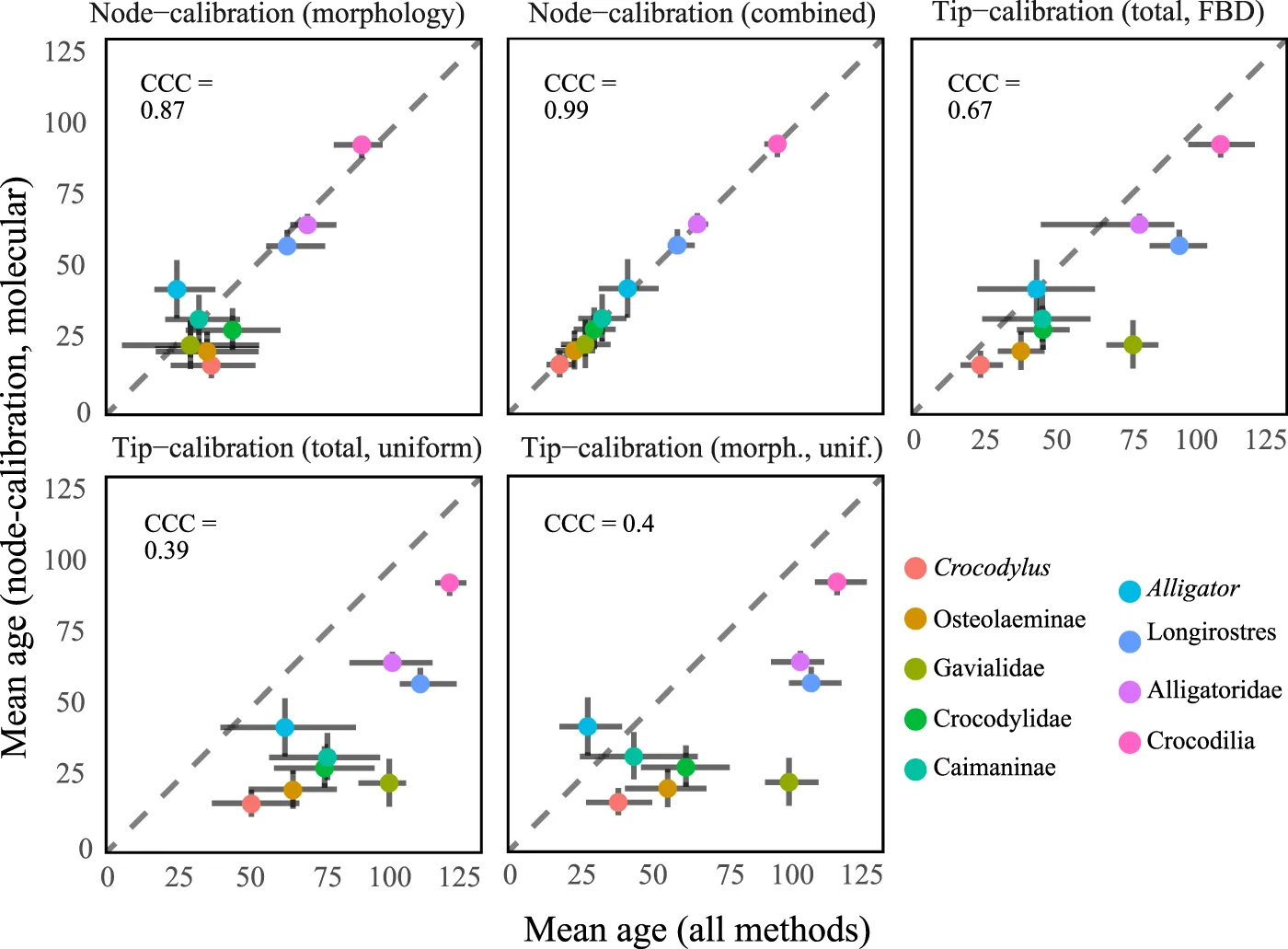
Node ages (Ma) from a node-calibrated (molecular) dating analysis plotted against posterior estimates using all other strategies. Point estimates represent mean ages, with horizontal and vertical bars depicting the 95% Highest Posterior Density intervals. For readability, results are truncated and only include major groups (see legend). CCC, concordance correlation coefficient.

### (c) Tip calibration under the uniform tree prior

Conditioned on the same extant tree topology as above, uniform tip-calibrated analyses (total-evidence and morphology-only) inferred a majority-rule (50%) consensus tree with limited resolution of fossil taxa and extensive polytomies (see supplementary material). Both datasets estimated an Early Cretaceous origin during the Aptian–Albian (total-evidence: 124.5–116.22 Ma; morphology-only: 121.41–102.18 Ma) (figure 3*d*,). Multiple higher-order clades were inferred to have evolved before the K–Pg boundary, including Longirostres (121.78–106.23 Ma; 114.5–93.52 Ma), Alligatoridae (116.36–86.01 Ma; 109.88–87.92 Ma) and Gavialidae (110.9–88.14 Ma; 106.79–84.39 Ma). Several other clades had HPD intervals spanning the Mesozoic–Cenozoic boundary, including crown Osteolaeminae (84.56–48.15 Ma; 67.45–36.92 Ma), Crocodylidae (110.29–64.16 Ma; 73.13–40.95 Ma) and crown Caimaninae (97.36–55.04 Ma; total-evidence only). The youngest major clade estimated in the total-evidence analysis was *Crocodylus* (69.28–35.41 Ma), whereas in the morphology-only analysis, it was *Alligator* (37.57–15.56 Ma). Older ages in the tip-calibrated analyses resulted in low concordance with the node-calibrated molecular timescale (CCC = 0.27; 0.44). Coefficients of variation were 0.41 and 0.52 and total tree lengths were 1247 and 948 Myr (total-evidence; morphology-only).

### (d) Tip calibration under the fossilized birth–death tree prior

Total-evidence analysis under the FBD process resulted in a better-resolved tree, although polytomies remained within some major clades in the consensus (e.g. Crocodylidae) (see supplementary material). The root divergence was dated to 118.1–94.4 Ma, spanning the Cenomanian and Aptian ages of the Early–Late Cretaceous (figure 3*f*). Longirostres (103.44–83.13 Ma) and Gavialidae (84.62–66.1 Ma) were estimated to have diverged prior to the K–Pg boundary, with Alligatoridae (87.56–41.26 Ma) spanning the Mesozoic–Cenozoic. All other divergences were estimated to occur during the Cenozoic, including crown Caimaninae (59.57–18.36 Ma), Crocodylidae (52.27–33.93 Ma), crown Osteolaeminae (43.55–27.6 Ma) and *Alligator* (62.24–20.83 Ma)*.* The youngest major clade estimated to diverge was *Crocodylus* (28.49–13.86 Ma), during the Miocene–Oligocene. These results were moderately concordant with the node-calibrated molecular timescale (CCC = 0.67), with a coefficient of variation and total tree length of 0.59 and 791 Myr, respectively.

## 4. Discussion

### (a) Do competing methods produce the same results?

To systematically compare calibration approaches, it is first essential to assess whether marginal (effective) time densities (a) are consistent with user-specified calibration densities and (b) meaningfully influence the resulting posterior estimates. Regarding the former, we ran a node-calibrated analysis without sequence data, confirming that marginal time densities on constrained nodes largely corresponded to user-specified calibration densities (figure 2). We extended this approach to tip-calibrated analyses, finding that marginal time densities significantly predated user-specified node calibrations, indicating that these two approaches are generating distinct, incompatible effective priors on node ages. Under a uniform prior, the marginal time densities resulting from tip calibration at times fall entirely outside the minimum and maximum bounds specified in node-calibrated analyses. FBD-based analyses impose a third set of prior expectations: clades with in-group fossils (including the root) have older marginal time densities, while fossil-poor clades exhibit younger node ages that extend toward the recent. Overall, divergence times sampled from the prior in our tip-calibrated analyses are older than both user-specified palaeontological constraints and marginal time densities resulting from node-calibrated analyses, although this pattern is not uniform across lineages under the FBD model.

The recovery of an older effective prior in tip-calibrated analyses is perhaps unsurprising owing to a lack of internal node-age constraints. In their absence, clade ages are estimated based on the distribution of fossil tips and the root calibration. Internal nodes must accommodate the uncertainty in terminal age ranges, skewing the distribution of prior probability toward increasingly ancient divergence times [48]. This phenomenon has been characterized as deep-root attraction (DRA) [18]. For each calibration strategy, the differences observed in effective priors propagate to posterior time estimates, resulting in three distinct evolutionary timescales for node calibration and tip calibration using uniform or FBD tree priors, respectively. Tip-calibrated node ages are inferred to be substantially older when analysed under a uniform tree prior, with both the origin and the emergence of major clades preceding the K–Pg boundary. Node calibration, by comparison, infers a much younger chronology wherein major diversification events occur predominantly in the Cenozoic. These differences strongly correlate with the structure of the marginal time densities observed in the effective prior. The FBD-based timescale diverges the most from its prior, with node ages frequently inferred to be more ancient in the posterior. Despite this, the overall pattern is the same: timescales inferred under the FBD process are intermediate to node- and uniform tip-calibrated chronologies, estimating the origin of major lineages throughout both the Mesozoic and Cenozoic. Collectively, these results suggest that inferred posterior timescales are highly sensitive to the structure of their underlying priors.

In addition to comparing calibration strategies, we also examined the impact of different datatypes by analysing molecular, morphological and combined datasets. For node-calibrated analyses, we inferred a timescale for three distinct dataset combinations (figure 3*a*,*b*,*c*). By contrast, tip-calibrated analyses yielded only two (figure 3*d*,*e*), because these analyses place fossil taxa alongside extant relatives based on morphological character data; therefore, there was no molecular-only analysis, although fossils are sometimes incorporated as tips into FBD analyses without morphological character data [49]. Within specific calibration frameworks, the resulting timescales closely mirrored broad-scale patterns observed in the prior, with minimal variation in the estimated root divergence across data partitions. This contrasts with the much larger discrepancies across competing calibration strategies. However, morphology-only datasets in both node- and tip-calibrated analyses show marked variation in the timing of shallower internal divergences relative to the root. For instance, both the morphology-based timescales (node and tip) place the radiation of *Crocodylus* prior to the divergence of *Alligator*—a scenario rejected by all model variants incorporating molecular data. Furthermore, morphology-only analyses (particularly under node calibration) produced markedly wider credibility intervals. Taken together, these results indicate that molecular and morphological datasets yield broadly comparable yet ultimately distinct reconstructions of crocodylian evolutionary history when analysed under separate calibration schemes. Nevertheless, the magnitude of these differences is far smaller than the disparities arising from calibration strategy, underscoring calibration choice (as implemented in the effective prior) as the primary driver of variation in divergence time estimation.

### (b) Which timescale is the most accurate?

Having demonstrated that different calibration strategies produce distinct timescales, our next step was to assess which model (if any) yields the most credible estimates. This is an important step because competing approaches can yield alternative scenarios of species diversification through time that carry distinct implications for interpreting evolutionary history. For example, our node-calibrated timescales suggest that the majority of crocodylian groups diversified after the K–Pg extinction event, coinciding with the emergence of oceanic seaways during the early Cenozoic [50]. Estimated using molecular data, this hypothesis infers comparatively high levels of diversification in the Neogene, a result previously obtained by [34]. Alternatively, uniform tip-calibrated approaches suggest a more ancient appearance of modern crocodiles, emerging in the Albian or Aptian ages of the Early Cretaceous, with interfamilial lineages radiating vicariantly against the backdrop of rifting between Eurasia and North America during the Late Cretaceous/Early Palaeocene [51]. Using this calibration strategy, a lower proportion of major clades was inferred to diversify during the Neogene. However, tip calibration under the FBD framework places the origin of more major clades across the Late Cretaceous and Palaeogene epochs, with Crocodylidae and ingroup alligatorids notably diversifying in the Neogene. To differentiate between these scenarios, we must first establish how accurately each dating method translates disparate lines of evidence into biologically plausible divergence times.

Determining which timescale is closest to the truth is inherently difficult, as true divergence times are unknowable for empirical datasets. Addressing this question requires the performance of each method to be evaluated relative to its underlying rationale—for example, node-calibrated timescales are inferred based on user-specified calibration densities representing qualitative assessments of the elapsed time between a clade's origin (maximum bound) and its oldest fossil record (minimum bound) [31]. In principle, node calibrations can serve as a benchmark for accuracy because they are based on explicit empirical justification. In practice, they are difficult to define objectively and their mis-specification can lead to erroneous or internally inconsistent chronologies [52]. Tip calibration (using both uniform and FBD tree priors) avoids this subjectivity by jointly estimating time and topology, while including all available fossil data [20]. In our analyses, tip calibration yielded substantially older divergence times, sometimes extending beyond the specified fossil maxima from node calibrations. This pattern suggests two possibilities: either tip-calibrated approaches are systematically overestimating divergence times, or some of the node calibrations themselves impose overly restrictive or inaccurate bounds. Distinguishing between these alternative scenarios requires a critical re-evaluation of key node calibrations to assess whether the clades in question could plausibly be as old as inferred under tip-calibrated analyses.

Maximum constraints on clade ages can be challenging to justify because they rely on negative evidence: an absence of fossils in a particular lineage does not definitively demonstrate that a clade had not yet evolved [11]. To account for this uncertainty, we applied hard maxima only when the fossil record provides strong evidence that older crown occurrences are improbable. For the root-age constraint, the maximum age was set to 95 Ma, an interval marked by the absence of crown-group fossils despite the presence of morphologically comparable eusuchians such as *Portugalosuchus* [35]. This dichotomy—stem crocodylians being preserved while crown forms are absent—indicates that the record is sufficiently complete that a lack of crown fossils is unlikely to reflect preservational or sampling biases, with the maximum comfortably predating the oldest unambiguous crown crocodylian (*Leidyosuchus*, 77–75 Ma). Consequently, this calibration is both permissive and defensible, with the much older crown origins suggested by tip calibration (particularly in analyses using a uniform tree prior) being improbable. For Alligatoridae, we applied a hard maximum of 83.4 Ma based on *Brachychampsa sealeyi*, the oldest crocodylian historically considered an alligatorid [53]. *Brachychampsa* derives from the Menefee Formation (81–75 Ma); accordingly, we set the maximum to a slightly older age to encompass an interval with suitable taphonomic conditions but no known crown material [54]. This approach is further supported by the high probability that *Brachychampsa* represents an early-diverging alligatorid, meaning its age is additionally unlikely to underestimate the true timing of the clade's origination [37]. Accordingly, the inflated timescales recovered by tip calibration, with an upper bound exceeding 100 Ma in analyses using a uniform tree prior, are more plausibly attributable to deep-root attraction than to overly restrictive node calibrations.

For nodes where the fossil record did not justify a hard maximum, we used offset-exponential calibration densities to approximate a soft-tailed distribution. This approach allocated a reasonable probability beyond the minimum constraint while avoiding an arbitrarily narrow upper bound. For example, the 95% HPD interval width for crown Caimaninae in the prior extended to 61.03 Ma, allowing for older placements of fragmentary alligatorid specimens (e.g. *Tsoabichi greenriverensis*) [37]. However, this did not encompass the earliest putative caimans such as *Orthogenysuchus olseni*, indicating this calibration may be somewhat restrictive, with the heterogeneous divergence estimates for this clade possibly reflecting their fragmentary fossil record and uncertain phylogenetic affinities rather than systematic bias or mis-specified calibration densities. In contrast, other offset-exponential calibrations behaved as intended. For example, the effective calibration for Longirostres allowed limited probability of a Mesozoic origin based on Late Cretaceous ‘thoracosaurs’, while still favouring a Cenozoic divergence consistent with current evidence that *Maroccosuchus zennaroi* and *Kentisuchus spenceri* represent the oldest fossil representatives for this clade (95% HPD approx. 83.5–53.9 Ma) [55,56]. Subsequently, the significantly older ages obtained from uniform tip-calibrated analyses, which suggest an Early Cretaceous origin for Longirostres, is unlikely to reflect mis-specified node constraints and is more readily attributable to systematic bias. The accuracy of tip calibration under the FBD prior, which produces a Late Cretaceous origin, is contingent on the putative affinity of thoracosaurs with the morphologically comparable gavialids; however, this hypothesis is yet to be corroborated by studies that integrate molecular evidence from extant species, with recent studies recovering thoracosaurs outside even Crocodylia [56,57].

An evaluation of user-specified node-age constraints demonstrates that both uniform and offset-exponential calibration densities—with the possible exception of Caimaninae—were sufficiently permissive to accommodate multiple competing scenarios of crocodylian evolution, while penalizing unrealistically older dates. Consequently, the substantially older ages recovered under uniform tip-calibrated analyses, often predating even poorly supported chronostratigraphic and phylogenetic hypotheses, are likely methodological artefacts resulting from deep-root attraction rather than overly restrictive or inaccurate node-age constraints. Using the FBD prior serves to mitigate, but does not eliminate, this tendency. On the other hand, estimated node-calibrated timescales, particularly when supplemented by molecular data, tend to produce chronologies sufficiently informed by the fossil record while simultaneously allowing for older divergences in cases of greater uncertainty. Overall, the congruence between calibration densities, posterior time estimates and the fossil record supports the node-calibrated analyses as the most accurate reconstructions of crocodylian evolutionary history. This supports the notion that modern crocodiles originated in the Late Cretaceous and radiated in the Palaeocene–Eocene, possibly via transcontinental dispersal in response to the establishment of marine corridors during the Early Cenozoic. Node-calibrated timescales utilizing molecular data additionally support the previously established notion that a high proportion of diversifications within Crocodylia (particularly in Crocodylidae and crown caimans) occurred during the Neogene, an outcome possibly correlative with the Miocene Climatic Optimum [34].

### (c) Deep-root attraction in tip-calibrated analyses

The systematic overestimation of species divergence times—that is, DRA—has been a consistent feature of tip calibration since its inception, with previous studies identifying a number of causes, including failure to account for diversified sampling, inadequate models of morphological evolution and unreasonable tree priors [18,58]. Our results emphasize that DRA is already inherent before the data are considered—subsequently, we consider DRA a feature of the prior, primarily resulting from an absence of internal node-age constraints [48]. This strongly influences the posterior, suggesting that the data are not sufficiently informative to overcome this conflict. Thus, the problem is twofold: the prior itself imposes a strong bias toward excessively deep ages, while the data themselves offer too weak a signal to correct the inherited bias. Regarding the former, implementing node and tip calibrations together has been shown to mitigate the propensity for tip-calibrated analyses to infer unfeasibly ancient divergence times: node calibrations constrain the uncertainty associated with tip calibrations, while tip calibrations reciprocally provide an objective basis for defining effective maxima on node ages [48,59]. While combined approaches are conceptually attractive, we did not implement a hybrid analysis in our study because MrBayes proposes ages for fossil tips that are incompatible with designated node calibrations, resulting in errors. Future research should therefore explore alternative implementations of combined tip- and node-calibrated strategies using a variety of software, in order to better utilize the complementary temporal signal provided by both calibration types.

Despite displaying the largest coefficients of variation in the prior, node calibration with molecular data inferred the most precise posterior timescale. These results suggest that the data are sufficiently informative for node ages to converge upon posterior time estimates that depart meaningfully from the prior. Contrastingly, node calibration with a morphology-only dataset inherited the large uncertainties of the prior, suggesting that phylogenetic signal is unevenly distributed across data partitions. This particular case has significant ramifications for tip calibration, in which temporal and topological accuracy are linked. If the data are uninformative, posterior estimates may not depart meaningfully from the prior toward younger, more realistic node ages [60]. In our study, coefficients of variation for uniform tip-calibrated timescales were largely unchanged in the posterior, and there were numerous unresolved nodes in the full tree (see supplementary material). Despite previous studies suggesting that inaccurate fossil placement in total-evidence analyses may not propagate to erroneous node-age estimates [61], our tip-calibrated morphology-only approach—a method increasingly used in the dating of entirely extinct clades—inferred a timescale that departed substantially from well established interpretations of crocodylian evolution based on molecular clocks, biogeography and the fossil record [27,57,62,63]. These outcomes highlight the acute difficulties of co-estimating time and tree topology from morphological data, even for a group with a comparatively well structured fossil record and numerous living representatives such as Crocodylia [64]. We advocate a cautious approach when applying morphological clock methods to fossil-only groups.

## 5. Conclusion

Overall, our findings demonstrate that strategy-specific priors exert a dominant influence in divergence time estimation. In our case study based on Crocodylia, we show that node-calibrated analyses estimate evolutionary timescales more compatible with the palaeontological, phylogenetic and stratigraphic evidence than competing chronologies derived from tip-calibrated approaches using both vague and mechanistic tree priors. This may hold for well sampled and well studied groups like Crocodylia, but deriving node calibrations remains challenging for clades characterized by sparse fossil sampling, uncertain phylogenetic placement or incomplete stratigraphic coverage. In such instances, we encourage researchers to explore competing dating methods and calibration strategies, evaluating the inferred timescales *post hoc* for plausibility in light of palaeontological and biogeographical evidence. Future research utilizing hybrid approaches may be promising in overcoming the limitations of individual dating strategies in order to obtain more accurate and precise evolutionary timescales across the tree of life.

## Supplementary Material



## Data Availability

Full analysis workflows, including datasets and MrBayes control files, have been deposited in the Figshare online open access repository [65]. Supplementary material is available online [66].
