# Supplementary material for: Competing methods of divergence time estimation recover diverse evolutionary timescales
Source: Proc Biol Sci. 2026 Oct 7;293(2080):20260635. doi: 10.1098/rspb.2026.0635 (PMC13641909; doi:10.1098/rspb.2026.0635)
Supplement: Supplementary file 1 [file rspb.2026.0635.zip › rspb.2026.0635/rspb20260635_si_001.zip!/supp/2.2_tip_calibrations.docx]

**Crocodilia Tip Ages**

**Acnyodon iberoccitanus | 66–69.05 Ma**

Specimens traditionally referred to as “*Acynodon iberoccitanus*” have been reported from several localities in Spain and France, although it is important to note that the material commonly included in phylogenetic datasets under this name does not represent *A. iberoccitanus*, nor even *Acynodon* (Turner & Brochu, in prep.). The holotype of *A. iberoccitanus* derives from Laño (Vitoria Formation, Castilla y León, Spain), a site dated to the Late Cretaceous on the basis of magnetostratigraphy and biochronostratigraphy. The Laño succession has been assigned a Late Campanian age in its lower part (*Hoplitoplacenticeras marroti* ammonite zone, correlated with Chron C33r), with the vertebrate site considered latest Campanian–early Maastrichtian, correlated with Chron C32n (ca. 72–73.5 Ma; Corral 2016). By contrast, the material typically included in phylogenetic analyses originates from Vitrolles (France), where the deposits are reported to be Late Campanian–Early Maastrichtian in age (see Prieto-Márquez 2013). The Spanish material is slightly younger. For temporal calibration purposes, placing the minimum bound at the base of the Maastrichtian is justified, whereas the upper bound may reasonably be set around the mid-Maastrichtian, here approximated as 66–69.05 Ma.

**Alligator mcgrewi | 15.9–17.5 Ma**

The holotype was recovered from Aletomeryx Quarry, Runningwater Formation (upper Marsland Formation), Cherry County, Nebraska (Schmidt 1941). Aletomeryx Quarry represents a terrestrial horizon within the Hemingfordian North American Land Mammal Age (NALMA). Although this horizon falls within the Burdigalian Stage of the Miocene, the NALMA provides a more precise temporal framework. The base of the Hemingfordian is placed at 18.9 Ma (Tedford et al. 2004), but the Aletomeryx Quarry is generally regarded as belonging to the upper Hemingfordian subinterval (He2), which spans 17.5–15.9 Ma (Tedford et al. 2004). This interval overlaps with the Burdigalian Stage (20.44–15.97 Ma), whose boundaries are defined by planktonic foraminiferal events and magnetochronology (Berggren et al. 1985; Gradstein et al. 2005; Lourens et al. 2005). Comparable calibrations for western North American *Alligator* species are discussed in Whiting and Head (2015).

**Alligator mefferdi | 10.3–12 Ma**

The holotype was recovered from the *Alligator mefferdi* Quarry (also known as George Sawyer Ranch), upper portion of the Ash Hollow Formation, Cherry County, Nebraska (Mook 1946). The *Alligator mefferdi* Quarry is assigned to the Clarendonian NALMA. Associated taxa from the site include the ungulate *Ustatochoerus major*, whose stratigraphic range extends from 13.6 to 7.25 Ma (Kent 1963). The mammalian assemblage as a whole supports a Clarendonian assignment, consistent with faunas of comparable age from Florida and Kansas (Diffendal 1982; Alroy 2000). The Clarendonian Stage is currently constrained to 13.6–10.3 Ma (Tedford et al. 2004), with the age of the *Alligator mefferdi* Quarry most likely falling within 12–10.3 Ma. Fossils from the Hemphillian NALMA of Florida tentatively referred to *A. mefferdi* (Snyder, 2007) would extend the upper bound of the species to as young as 4 Ma, but this material was not considered in this analysis. Comparable calibrations for western North American *Alligator* species are reviewed in Whiting and Head (2015).

**Alligator olseni | 15.9–18.9 Ma**

The holotype was recovered from Thomas Farm, near Bell, Gilchrist County, Florida (White 1942). Thomas Farm has traditionally been assigned to the early Hemingfordian (He1) NALMA, based on the co-occurrence of *Phoberocyon* (Ursidae), *Leptarctus* (Mustelidae), *Floridaceras* (Rhinocerotidae), and *Metatomarctus* (Canidae) (Tedford et al. 2004). These taxa are also present in Early Miocene faunas of the western United States, dated independently by radiometric and paleomagnetic methods (Tedford et al. 2004). Thomas Farm contains at least four taxa listed by Tedford et al. (2004) as not surviving beyond He1: *Floridaceras, Menoceras, Phoberocyon*, and Anthracotheriidae. Tedford et al. (2004) also noted several genera first appearing in the He2 subinterval, including *Petauristodon*, which is represented at Thomas Farm. However, *Petauristodon* has also been documented from He1 faunas, such as the Centenario Fauna of Panama (MacFadden 2014), suggesting that its dispersal into North America occurred by He1 or earlier. Therefore, its occurrence at Thomas Farm does not necessitate a He2 age. The Thomas Farm locality is generally correlated with the Hemingfordian NALMA (18.9–15.9 Ma; Tedford et al. 2004), which broadly overlaps with the Burdigalian Stage of the Miocene (20.44–15.97 Ma), defined by planktonic foraminiferal events and magnetochron boundaries (Berggren et al. 1985; Gradstein et al. 2005; Lourens et al. 2005).

**Alligator prenasalis | 33.9–37.2 Ma**

The holotype was recovered from the Chadron Formation, Indian Draw, Pennington County, South Dakota (Loomis 1904). The Chadron Formation has been assigned to the Chadronian NALMA, based on its characteristic mammalian fauna. The upper boundary of the Chadronian within the White River Group has also been defined by the first appearance of *Hypertragulus calcaturus* at 33.7 Ma (Prothero 1998). The formation contains mammalian assemblages characteristic of the Chadronian, including the Titanothere Zone of the Lower White River Beds (Mehl 1916). Magnetostratigraphic correlation places the formation within Chrons C12 and C13, and radiometric (K–Ar) dates further constrain its age (Berggren et al. 1985). These data indicate correlation with the Priabonian Stage of the late Eocene. The base of the Priabonian is defined by the first occurrence of the calcareous nannoplankton *Chiasmolithus oamaruensis*, which marks the base of nannoplankton biozone NP18 and is dated at 37.2 Ma. The top of the Priabonian is defined by the extinction of the foraminiferal genus *Hantkenina*, dated at 33.9 Ma. Alligator prenasalis has been reported from the overlying Brule Formation, which extends into the Orellan NALMA (33.9 Ma – 32 Ma) (Whiting and Hastings, 2015) but pending review of this material, it was not considered here.

**Allodaposuchus precedens | 66.0–71.5 Ma**

The specimen was recovered from the Tuştea–Oltoane nesting site, Densuş-Ciula Formation, Hunedoara, Romania, and from Pui 4, Sibişel Valley, Sânpetru Formation, Hunedoara, Romania. Both formations have been dated to the Maastrichtian. The Sânpetru Formation has been assigned an Early Maastrichtian age based on palynological evidence (Van Itterbeeck 2005) and magnetostratigraphic studies (Panaiotu & Panaiotu 2010). The pollen assemblage from the Pui site correlates with that of the Densuş-Ciula Formation, suggesting that the two are of equivalent age. The palynological assemblage of the Maastrichtian stratotype shares 23 angiosperm species with the Romanian sites, supporting an early Late Maastrichtian age (Van Itterbeeck 2005; Jagt 2002). Paleomagnetic data further constrain the age of these formations between the base of Chron C32n.1n and the top of Chron C31n (i.e., 71.5–68.7 Ma), an interval that lies within the Maastrichtian (Panaiotu & Panaiotu 2010). The upper boundary of the Maastrichtian is defined by the Cretaceous–Paleogene (K–Pg) boundary at 66 Ma, marked by a mass extinction of flora and fauna, a global iridium anomaly, and radiometric dating (Renne et al. 2013).

**Allognathosuchus polyodon | 47.5–51.5 Ma**

*Allognathosuchus polyodon* is known with certainty only from Grizzly Buttes, Lower Member of the Bridger Formation, Uinta County, Wyoming. Radiometric dating (K–Ar) has yielded an age of 50.4–50.1 Ma for this site (Krishtalka & Stucky 1987), with subsequent calibration placing the upper boundary at 50.5 Ma by correlation with Chron C22r (Clyde et al. 1997). The species has been reported from elsewhere in deposits of Bridgerian and Uintan age. The type and referred specimens of *A. polyodon* are best constrained to the Bridgerian NALMA, which spans approximately 51.5–47.5 Ma (Woodburne 2004; Robinson et al. 2004), overlapping the Ypresian–Lutetian boundary. While some recent estimates place the upper bound around 47.5 Ma (entering the lowermost Lutetian), this may ultimately be revised downward to the end of the Ypresian. For present purposes, a range of 51.5–47.5 Ma is adopted.

**Allognathosuchus wartheni | 50.3–56.8 Ma**

Specimens have been recovered from several localities in Wyoming, including Tatman Mountain (Willwood Formation). These sites have variably been assigned to the Wasatchian and Clarkforkian NALMAs, spanning an interval between 56.0 and 50.3 Ma. The type material of this taxon is Wasatchian in age. Age determinations are based primarily on mammalian biochronology correlated with magnetostratigraphy. Clarkforkian faunas occur within strata deposited during a normal polarity interval correlated with Chron C25n, whereas overlying reversed polarity strata containing early Wasatchian faunas are correlated with Chron C24r (Berggren et al. 1985).

**Argochampsa krebsi | 61.7–66.0 Ma**

Specimens have been recovered from several localities in Morocco, including the Rhinopolis collection and the Oulad Abdoun Basin, Grand Daoui area (Hua 2004). These deposits have been dated to the Danian–Thanetian boundary (Paleocene) on the basis of faunal assemblages, with correlation to Danian strata at El Kef, Tunisia. The primary criterion for recognition of the Cretaceous–Paleogene (K–Pg) boundary is the global iridium anomaly, interpreted as the result of an extraterrestrial impact and associated with major biotic turnover, including the extinction of ammonites, restructuring of nannoplankton communities, and the demise of non-avian dinosaurs (Hildebrand et al. 1991; Luterbacher et al. 2005). This iridium spike has been dated to 66 Ma by magnetostratigraphy and cyclostratigraphy (Husson et al. 2011) as well as isotopic methods (Renne et al. 2013). The top of the relevant interval is defined by planktonic foraminiferal and calcareous nannoplankton biostratigraphy, notably the first appearance of several *Fasciculithus* species and the earliest occurrence of *Neochiastozygus perfectus*, yielding an age of approximately 61.7 Ma (Stainforth et al. 1975).

**Asiatosuchus germanicus | 47.01–48.06 Ma**

Specimens have been reported from Geiseltal (Mittelkohle and Unterkohle), Sachsen-Anhalt, Germany, but more reliably from Messel, which provides the most secure age constraint. The holotype is from Messel, and codings for the species in phylogenetic analyses are based on specimens from that locality. The Messel deposits are dated to the Lutetian. The base of the stage is defined by the first appearance of the calcareous nannofossil *Blackites inflatus* (Blondeau 1981). The top is defined by the first appearance of *Reticulofenestra reticulata*, correlated with the C19n/C18r boundary and dated at approximately 41.2–41.3 Ma (Marino & Benjamini 2002). If restricted to Messel material, *A. germanicus* is dated to ca. 48–47 Ma. Recent chronostratigraphic work on the Messel sequence (Kaboth-Bahr et al. 2023) supports this narrower age assignment, corresponding to the middle Eocene (early Lutetian).

**Asiatosuchus nanlingensis | 59.2–61.5 Ma**

Specimens have been recovered from several sites within the Zhuguikou Member of the Nongshan Formation, Guangdong, China. The Nongshan Formation is correlated with the Middle Paleocene (Selandian) and assigned to the Nongshanian Asian Land Mammal Age (ALMA), dated to 61.5–59.2 Ma (Clyde et al. 2008; Zachos et al. 2018).

**Australosuchus clarkae | 23.0–26.0 Ma**

Specimens attributed to *Australosuchus clarkae* have been recovered from several localities, including Lake Pinpa and Lake Tarkarooloo (Namba Formation) and the Tedford locality, Lake Palankarinna (Etadunna Formation), South Australia. The Namba Formation has been considered Middle Miocene in age, based on palynological assemblages and mammalian fauna, particularly vombatoid marsupials (Rich & Archer 1979). Its pollen floras closely resemble those from Batesfordian–Balcombian deposits in Victoria (Muddy Creek Marl) and South Australia (Munno Para Clay), supporting a Middle Miocene age of ca. 14–16 Ma for vertebrate assemblages occurring near the top of the Namba Formation (Tedford et al. 1977). The Etadunna Formation, by contrast, has been more securely dated to the latest Oligocene–earliest Miocene. Its distinctive dolomitic mudstones and illitic–smectitic clays with palygorskite, combined with faunal correlations, indicate equivalence with other Australian formations of Chattian to Burdigalian age (Woodburne et al. 1993). Biostratigraphic correlation places the base of the Etadunna in the Chattian (defined by the extinction of *Chiloguembelina* and the base of planktonic foraminiferal biozone P21b, ca. 28.1 Ma) and its top no younger than the Burdigalian (defined by the first appearance of *Praeorbulina glomerosa* and the top of Chron C5Cn.1n, ca. 15.97 Ma; Depéret 1892). However, more recent stratigraphic constraints restrict the Etadunna Formation to the latest Oligocene (26–23 Ma), with the younger bound likely reflecting assemblages from the Namba Formation (Woodburne et al. 1994). Most published specimens of *A. clarkae* are derived from the Etadunna, and this narrower interval (26–23 Ma) is therefore adopted here.

**Borealosuchus acutidentatus | 61.7–66.0 Ma**

Specimens have been recovered from Montague Lake, Ravenscrag Formation, Saskatchewan, Canada. The Ravenscrag Formation spans the Cretaceous–Paleogene (K–Pg) boundary, dated by palynological and lithological evidence. The boundary is marked by a geochemical anomaly in iridium levels and the presence of shocked quartz, both associated with the mass extinction event. At this site, the extinction is reflected in the disappearance of pollen and spore taxa characteristic of Late Cretaceous floras, followed by an abundance of fern spores, the so-called “fern spore spike” (Fleming & Nichols 1990). The Ravenscrag Formation is conventionally considered to terminate at the end of the Puercan, ca. 66 Ma, by correlation with the Puerco fauna of New Mexico (Sternberg 1932). Specimens have also been reported from Kimbetoh Arroyo, Nacimiento Formation, San Juan County, New Mexico, but these were not considered here pending further review.

**Borealosuchus formidabilis | 56.8–60.2 Ma**

*Borealosuchus formidabilis* is known with certainty from a single locality, Wannagan Creek, North Dakota, which provides the basis for terminal codings in phylogenetic analyses. Wannagan Creek is assigned to the Tiffanian NALMA, dated between 60.2 and 56.8 Ma (Rose 1981). For this reason, the species is here constrained to the narrower interval of 60.2–56.8 Ma. Specimens referred to *B. formidabilis* have been reported from several other localities across the USAincluding the Clarkforkian of Wyoming and Campanian of North Carolina, but these are currently spurious.

**Borealosuchus sternbergii | 63.6–68.2 Ma**

Specimens of *Borealosuchus sternbergii* have been reported from several localities in the USA, including South Dakota, North Dakota, Montana, Wyoming, and Colorado. The species is best documented from the Hell Creek and overlying Tullock formations. The Tullock Formation (e.g., UCMP V-78020, Garfield County, Montana) has been dated to the Puercan North American Land Mammal Age (NALMA). The Cretaceous–Paleogene (K–Pg) boundary within the formation is marked by distinctive coal deposits, an iridium anomaly, and the highest occurrences of Cretaceous pollen. Magnetostratigraphic calibration places the boundary between Chrons C28n and C29r, dated to 63.6–64.7 Ma (Swisher et al. 1993). The Hell Creek Formation provides the lower bound for *B. sternbergii*. Lithologic and biostratigraphic evidence firmly place this unit within the Maastrichtian. Recent work constrains its base to no older than 68.2 Ma (Fowler 2018), with no evidence supporting occurrences in the early Maastrichtian. Thus, the stratigraphic range of *B. sternbergii* is restricted here to 68.2–63.6 Ma.

**Borealosuchus wilsoni | 51.5–55.8 Ma**

The holotype of *Borealosuchus wilsoni* was recovered from the Green River Formation, Wamsutter, Sweetwater County, Wyoming. The Green River Formation was deposited over a ca. 5 Myr interval, spanning Chrons C24n through C21r, and dated to approximately 53.5–48.5 Ma (Smith et al. 2003). The vertebrate fauna of the Green River Formation is among the most complete records for the Late Wasatchian–Early Bridgerian North American Land Mammal Ages (NALMAs), with biochronological resolution provided by the first appearances of several primate and perissodactyl taxa (Stucky 1990). The type specimen of *B. wilsoni* derives from the Wasatchian Fossil Lake deposits of the Green River Formation, which correspond to the later part of the Wasatchian. In the absence of more precise stratigraphic constraints, the age of *B. wilsoni* is conservatively taken as 55.8–51.5 Ma, equivalent to the range of the Wasatchian. Much younger specimens were

**Boverisuchus vorax | 47.5–51.5 Ma**

The holotype of *B. vorax* is probably from the Bridger B (middle Bridgerian) at the Grizzly Buttes locality, while a well-preserved skull housed at the Field Museum in Chicago from the lower Washakie Formation, corresponding to the later Bridgerian (Brochu, 2012). The Bridgerian has traditionally been considered part of the middle Eocene (Lutetian), but revised stratigraphic and radiometric calibrations now place most of it in the early Eocene (Ypresian). The Bridgerian interval is magnetostratigraphically correlated with Chrons C23n–C22r and radiometrically constrained (^40Ar/^39Ar) to approximately 51.5–47.5 Ma (Clyde et al. 2000; Berggren et al. 1985). Accordingly, the stratigraphic range of *B. vorax* is here taken as 51.5–47.5 Ma. *Boverisuchus* is also known from sites in southern and western Texas, but these probably do not pertain to *B. vorax*.

**Brachychampsa montana | 66.0–68.2 Ma**

Specimens of *Brachychampsa montana* have been reported from several localities across the United States, including New Mexico, Wyoming, North Dakota, Montana, South Dakota, and Colorado. The species is reliably known only from the Hell Creek Formation, which spans the latest Maastrichtian. The K–Pg boundary is well defined in this unit by the iridium anomaly, shocked quartz, and the extinction of characteristic Cretaceous taxa, dated precisely to 66.0 Ma (Renne et al. 2013). Recent work places the base of the Hell Creek Formation at no older than 68.2 Ma (Fowler 2018). Earlier records from the Allison Member of the Menefee Formation (New Mexico), correlated with the Early Campanian (83.5–72.1 Ma) (Hunt 1993), and other Campanian material reflect misidentifications or taxonomic conflation with *Brachychampsa sealeyi* (Lucas & Sullivan). These occurrences are not attributable to *B. montana* and are excluded from the calibration range. Accordingly, the stratigraphic range of *B. montana* is restricted to 68.2–66.0 Ma.

**Brochuchus pigotti | 17.0–20.0 Ma**

Specimens of *Brochuchus pigotti* have been recovered from Kenya. The youngest occurrences are from Shackleton Gully, Fossil Bed Member, Hiwegi Formation, Rusinga Island, Kenya. The Hiwegi Formation has traditionally been assigned an age of ~17.8 Ma (early Miocene), though more recent geochronological analyses suggest that the Fossil Bed Member may be older than 18 Ma (Peppe et al. 2011; Conrad et al. 2013). Broader age constraints for the Hiwegi Formation have been derived from the Rusinga Agglomerate and Lunene Lava, yielding 19.6–16.5 Ma (Van Couvering 1969). Specimens reported from Saudi Arabia are not referrable to *B. pigotti*.

**Ceratosuchus burdoshi | 55.8–56.8 Ma**

The holotype of *Ceratosuchus burdoshi* was recovered from Rainbow Valley, Park County, Wyoming. This locality has been biochronostratigraphically dated to the Clarkforkian North American Land Mammal Age (NALMA), spanning 56.8–55.8 Ma. Rainbow Valley is one of the principal sites for the Phenacodus–Ectocion Zone, which represents the late Clarkforkian. This zone is characterized by the first appearances (FO) of *Esthonyx grangeri* and *Phenacolemur praecox*, and by the absence of *Phenacolemur cookei* (Rose 1981).

**Crocodylus affinis | 47.5–51.5 Ma**

The holotype of *Crocodylus affinis* was recovered from the Green River Formation, Wyoming, USA. This formation was deposited over ~5 Myr, spanning Chrons C24n through C21r, and dated to 53.5–48.5 Ma (Smith et al. 2003). The Green River Formation is also biochronologically well constrained, preserving one of the most complete faunal records across the Late Wasatchian–Early Bridgerian North American Land Mammal Ages (NALMAs). These faunas are characterized by the first appearances (FA) of several primate and perissodactyl taxa (Stucky 1990). Although crocodylian remains from older (Wasatchian) and younger (Uintan) horizons in the Western Interior have sometimes been treated informally as *Crocodylus affinis*, these specimens are incomplete and have not been included in published phylogenetic codings. Accordingly, the stratigraphic range of *C. affinis* is here conservatively taken as 51.5–47.5 Ma.

**Crocodylus anthropophagus | 0.6–1.9 Ma**

Specimens have been found in multiple localities across Tanzania. The holotype was collected at Crocodile Korongo (Beds III–IV), CROC K, Olduvai Formation, Tanzania. It was recovered on the surface between two tuffs dated to approximately 1.84 million years ago (Ma), within the same interval as the type localities for the hominins *Homo habilis* and *Australopithecus boisei* (Brochu 2010). The fossiliferous sequence of the Olduvai Formation was deposited entirely before the Brunhes/Matuyama boundary, ca. 0.78 Ma (Tamrat 1995). The discovery of a reversed magnetozone in the Masek Beds (R3) indicates that the Olduvai Formation was deposited during the Matuyama chron, corresponding to the Gelasian–Calabrian, Lower Pleistocene. This interval magnetostratigraphically spans Chrons C2r–C1r, i.e. 2.6–0.9 Ma (Lourens 2005). Based on current evidence, the oldest known specimens are dated to ca. 1.9 Ma, while the youngest specimens are from Bed IV, placing the upper bound at ca. 0.6 Ma.

**Crocodylus megarhinus | 28.4–33.9 Ma**

Specimen found in Birket-el-Qurun, Upper Fossil Wood Zone, Jebel Qatrani Formation, Fayum, Egypt. The Jebel Qatrani Formation has been dated to the Priabonian–Rupelian (Late Eocene–Early Oligocene) by lithostratigraphy and correlation with molluscan and foraminiferal faunas (Gingerich 1993). The Eocene–Oligocene boundary is attributed to the Lower Fossil Wood Zone (Rasmussen 1992), so only the Rupelian boundary is considered for the Upper Fossil Wood Zone. The base of the Rupelian is defined by the extinction of hantkeninid planktonic foraminifera, which corresponds with nannofossil zone NP21 and Chron 13r.1. The Eocene–Oligocene boundary has also been dated radiometrically (K–Ar and Ar–Ar) to ca. 34 Ma (Premoli-Silva & Jenkins 1993). The top of the Rupelian is defined by the extinction of the genus *Chiloguembelina* and the base of foraminifer biozone P21b (Luterbacher 2005).

**Crocodylus palaeindicus | 2.6–12.0 Ma**

Specimens found in Pakistan and India. The earliest occurrence is in the Daud Khel Local Fauna, Chinji Formation, Siwalik Group, Minanwali, Pakistan. The Chinji Formation’s boundaries have been placed between Chrons C15 and C10, i.e. 14.3–10.8 Ma, using stratotype data (Johnson 1985). The latest occurrence is in Punjab, India, Upper Member of the Siwalik Formation. This locality has been dated to the Pliocene, Piacenzian. The top of the Piacenzian is defined by the base of the Matuyama (C2r) chron, above which occur the extinctions of the nannofossils *Discoaster pentaradiatus* and *Discoaster surculus*, at 2.59 Ma (Lourens et al. 2005).

**Crocodylus thorbjarnarsoni | 1.5–4.22 Ma**

Specimens found in several localities across Kenya. The oldest occurrence is in Lothagam, Apak Member, Nachukui Formation, Kenya. The boundary between the Lothagam-Upper Nawata Member and the Apak Member has been K–Ar dated to 4.22 ± 0.03 Ma (Feibel 2003). The youngest occurrence is in East Turkana, areas 105 and 102, Koobi Fora Formation, Kenya. Area 105 is from below the KBS Tuff, dated radiometrically to 1.9 Ma, although the section extends upward into younger deposits. The base of Area 102 is no older than 2.0 Ma, and the vertebrate fossils are found in horizons predating the Koobi Fora Tuff, which is radiometrically dated to 1.5 Ma. This places the specimens within an age range of ~1.5–2.0 Ma (Brochu & Storrs 2012).

**Diplocynodon darwinii | 47.41–48.25 Ma**

Holotype found in the Messel Formation, Hessen, Germany. The formation was deposited in a meromictic maar lake environment, which formed as a result of phreatomagmatic eruption(s). The Messel Pit is a well-known Lagerstätte, strongly correlated with the Ypresian/Lutetian (Lower/Middle Eocene) boundary. A revised ^40Ar/^39Ar date for the initial eruption(s) at Messel suggests an age for the beginning of crater formation between 48.5 and 47.9 Ma. Accordingly, the Messel Maar Formation must have been deposited during a 640 ka interval between 47.0 and 48.3 Ma. Astronomical dating has been tested for this time interval in correlation with palynological calibration. The best match of the pollen record of the Messel sequence with the orbital solution La2010a corresponds to the interval 47.41–48.05 Ma, whereas the astronomical calibration using La2010d suggests 47.61–48.25 Ma (Lenz 2015). Since the authors could not determine which method was superior, the joint time interval of 47.41–48.25 Ma is accepted.

**Diplocynodon hantoniensis | 36.0–39.5 Ma**

Specimens found in the Hordle Cliff Crocodile Bed, Lower Headon Beds Formation, Hampshire, England. The Crocodile Bed has been dated to the Upper Eocene, as it lies above the Leaf Bed and almost immediately below the upper ironstone band (Wood 1882), and is further supported by correlation with the mammal fauna (British Museum, 1973). The fossil record falls within the ELMA zone MP17, considered to span 39.5–36.0 Ma (Holman 2005).

**Diplocynodon remensis | 56.0–57.2 Ma**

Specimen found in Mont Berru, Sables de Bracheux Formation, Champagne-Ardenne, France. Mont Berru has been dated to the Late Paleocene, with its land mammal fauna assigned to MP5, i.e. early Thanetian (Hooker 1996). The lower bound is set at the Paleocene–Eocene boundary (56.0 Ma), which is defined by a pronounced negative carbon isotope excursion and a major turnover of deep-sea benthic foraminifera, calcareous nannoplankton, and mammals (Luterbacher et al. 2005). The upper bound (57.2 Ma) reflects the top of magnetochron C26n, which is conventionally correlated with the upper limit of MP5 in European Land Mammal Age stratigraphy (Hooker 1996; Luterbacher et al. 2005).

**Eocaiman cavernensis | 36.0–39.2 Ma**

The holotype was recovered from the Notostylops Beds, Sarmiento Formation, Chubut, Argentina. The Sarmiento Formation overall has been magnetostratigraphically dated to span the Middle Eocene through Early Miocene interval (~41.7–18.8 Ma) (Ré 2010). The Notostylops Beds were correlated with the Casamayoran/Barrancan South American Land Mammal Age (SALMA) (Bellosi 2010). Earlier estimates placed the Barrancan at ~42.0–37.5 Ma (Woodburne 2014). More recently, Godoy et al. (2020) revised the stratigraphic range of *Eocaiman cavernensis*, asserting that the Barrancan should instead be constrained to ~41.6–39.0 Ma. Based on this newer calibration, the stratigraphic range of *E. cavernensis* is updated here to 39.2–36.0 Ma (i.e. late middle Eocene).

**Eogavialis africanus | 33.9–37.2 Ma**

The holotype was recovered from the Beadnell Section, Qasr el-Sagha Formation, Egypt. The Qasr el-Sagha Formation has been biochronostratigraphically dated to the Priabonian, based on the occurrence of marker taxa such as *Saghacetus osiris* (Seiffert 2008). The Priabonian Stage spans 37.2–33.9 Ma. Its base is defined by the first appearance of the calcareous nannoplankton *Chiasmolithus oamaruensis*, which marks the base of biozone NP18, while its top is defined by the extinction of the planktonic foraminifer genus *Hantkenina* (Luterbacher et al. 2005; Gradstein et al. 2012).

**Eosuchus lerichei | 55.2–59.2 Ma**

The holotype was collected from Jeumont, in the Dinoflagellate cyst zone of the Grandglise Sand Member, Hannut Formation, France. The Grandglise Sand Member represents the youngest deposit of the Hannut Formation and has been dated to the Early Landenian (Thanetian) on the basis of lithostratigraphy, palynology, and carbon isotope data (Magioncalda et al. 2003). The upper boundary of the Thanetian, at 59.2 Ma, is defined by the base of magnetic Chron C26n and the first and last occurrences of several key nannofossil and dinocyst taxa (Luterbacher et al. 2005). The deposits preserving the holotype have been correlated with NP8, with associated estimates spanning MP8–MP9, yielding an approximate age range of 56.2–50.8 Ma. However, given stratigraphic uncertainties, a conservative estimate of 56.2–59.2 Ma is applied here (Magioncalda et al. 2003; Luterbacher et al. 2005). Fossils referable or possibly referable to *Eosuchus* occur in Eocene deposits on both sides of the Atlantic. Material from the early Eocene London Clay (UK) is clearly referable to the genus, though not necessarily to *E. minor* or *E. lerichei*.

**Eosuchus minor | 56.0–59.2 Ma**

Specimens found in several localities across the USA, including Maryland, Virginia, and New Jersey. The earliest occurrences are in the Aquia Formation in Maryland and Virginia. This formation has long been debated due to controversial stratigraphical and fossil evidence, but it is now dated to the Late Paleocene (Landenian, Thanetian). The Late Paleocene age is indicated by the presence of taxa such as *Ceratobulimina perplexa*, *Citharina plumoides*, *Robulus midwayensis*, and *Bulimina cacumenata* (Page 1959). The Thanetian upper boundary at 59.2 Ma is defined by magnetic Chron C26n, as well as the FO of the coccolithophore *Areoligeria gippingensis* and the LO of *Palaeocystodinium bulliforme* (Luterbacher 2005). The latest occurrences are in the Vincentown and Manasquan Formations of New Jersey, which are dated to the Late Paleocene. The Manasquan Formation correlates with calcareous nannofossil zones NP9–NP10 through biochronostratigraphy (Gibson 1993). Based on these correlations, *E. minor* is restricted to the Paleocene, with an age range of 56.0–59.2 Ma (Self-Trail et al. 2023).

**Eothoracosaurus mississippiensis | 70.0–76.0 Ma**

Specimens found in Tennessee and Mississippi, USA. The earliest occurrence is in the Coon Creek Tongue Member, Ripley Formation, McNairy County, Tennessee. The Coon Creek Tongue Member has been extensively studied and is dated to the Late Cretaceous, straddling the Campanian–Maastrichtian boundary. The gastropod fauna and lithology were first correlated with nearby formations in Tennessee and Mississippi (Wade 1926). The foraminiferal assemblages are exceptionally well preserved and, together with petrological data, support an early Maastrichtian age (Berry 1929; Mancini 1989). Ammonite fossils, including *Cirroceras conradi*, *Hoploscaphites reesidei*, *Solenoceras reesidei*, *S. texanum*, and *Nostoceras (N.) hyatti*, indicate an older upper Campanian bound, dated to 71.5–72.0 Ma by ^40Ar/^39Ar analyses (Larson 2012). Palynological evidence also supports a Late Cretaceous, early Maastrichtian age (Baghai-Riding 2016). Taken together, these data constrain the range of *Eothoracosaurus mississippiensis* to 76.0–70.0 Ma, with the type specimen from Mississippi representing early Maastrichtian deposits and Coon Creek specimens from Tennessee extending the range downward into the latest Campanian.

**Euthecodon arambourgi | 17.0–19.0 Ma**

Holotype found in Gebel Zelten site 6412, Maradah Formation, Libya. The Gebel Zelten assemblages have been assigned a Lower Miocene (Burdigalian) age, constrained between ~19 and 17 Ma, based on biochronostratigraphy of small mammal faunas (lagomorphs and cricetids) representing different evolutionary stages in North Africa (Fejfar & Horáček 2006). Crocodylian remains, including *Euthecodon arambourgi*, have been described from the same deposits, further supporting a Burdigalian age assignment (Llinás Agrasar 2004).

**Gavialis lewisi | 5.3–3.5 Ma**

Specimens are from the Dhok Pathan Formation, Middle Siwalik Group, Punjab, India. The Dhok Pathan succession spans the Late Neogene and has been dated using sedimentology, magnetostratigraphy, and correlations to Siwalik mammal faunas (Barry et al. 2002). For calibration, occurrences attributable to *G. lewisi* are restricted here to the Pliocene portion of the section. The lower bound is set at the base of the Pliocene (Zanclean) at ~5.33 Ma, and the upper bound near the early Piacenzian (~3.6–3.5 Ma), following the standard stage definitions (Lourens et al. 2005).

**Gavialosuchus eggenburgensis | 18.7–20.5 Ma**

Holotype found in Eggenburg, MN 3 mammal zone, Burgschleinitz Formation, Austria. The Burgschleinitz Formation is Miocene in age, with the MN3 mammal zone corresponding to the Upper Eggenburgian, Burdigalian. The Eggenburgian Stage is defined by the first appearances (FADs) of Loibersdorf-type mollusks (*Chlamys gigas* and *Pitar lilacionides*), as well as the benthic foraminifera *Elphidium ortenburgense* and *Uvigerina posthantkeni*, giving an age of 20.5–18.7 Ma (Steininger & Senes 1968).

**Gryposuchus colombianus | 11.6–13.8 Ma**

Specimens found in several localities in Colombia and Peru. One occurrence is from Duke Locality 79, La Venta Fauna, La Victoria Formation, Honda Group, Colombia. The Honda Group has been dated by ^40Ar/^39Ar to 13.8–11.6 Ma, and is magnetostratigraphically correlated with Chrons C5An.1n–C5ABn. In addition, the La Venta mammal fauna has been correlated with the Friasian stage of the SALMA (Flynn 1997).

**Gryposuchus croizati | 5.3–11.6 Ma**

Specimen found in the Middle and Upper Members of the Urumaco Formation, Falcón, Venezuela. The Urumaco Formation lies within the *Echitricolporites spinosus* Zone (Late Miocene) (Hambalek 1994). Mammal assemblages in this formation date it to the Tortonian and Messinian (Sánchez-Villagra 2010; MacFadden 2006). The base of the Tortonian is defined by the last common appearance of the nannoplankton *Discoaster kugleri* and the planktic foraminifer *Globigerinoides subquadratus*, associated with chron C5r.2n, dated to 11.6 Ma. The top of the Messinian is defined by the appearance of the nannoplankton *Ceratolithus acutus* and chron C3r, dating it to 5.3 Ma (Lourens 2005).

**Hassiacosuchus haupti | 47.41–48.25 Ma**

Holotype found in the Messel Pit, Germany. The formation was deposited in a meromictic maar lake environment formed by phreatomagmatic eruption(s). The Messel Pit is a well-known Lagerstätte, strongly correlated with the Ypresian/Lutetian (Lower/Middle Eocene) boundary. Revised ^40Ar/^39Ar dates for the initial eruptions at Messel suggest crater formation began between 48.5 and 47.9 Ma. Accordingly, the Messel Maar Formation must have been deposited during a 640-ka interval between 47.0 and 48.3 Ma. Astronomical dating, correlated with palynological calibration, indicates two possible intervals: 47.41–48.05 Ma (La2010a orbital solution) and 47.61–48.25 Ma (La2010d orbital solution). As the authors could not determine which solution was superior, the joint time interval of 47.41–48.25 Ma is accepted (Lenz 2015).

**Iharkutosuchus makadii | 83.6–86.3 Ma**

Holotype found in the Jókai mine, Veszprém, Csehbánya, and Ajka formations, Hungary. These formations are contemporaneous and have been dated to the Santonian (Upper Cretaceous) based on biochronology and geochemical studies, including analyses of *Iharkutosuchus* teeth (Kocsis 2009). The base of the Santonian is defined by the first occurrence (FO) of the inoceramid bivalve *Platyceramus undulatoplicatus*, while the top is defined by the last occurrence (LO) of the crinoid *Marsupites testudinarius* (Ogg 2005).

**Ikanogavialis gameroi | 5.3–11.6 Ma**

Specimens found in the Socorro and Urumaco formations, Falcón, Venezuela. The earliest occurrence is from Quebrada Honda, Socorro Formation, which has been dated to the Middle–Late Miocene through palynological and foraminiferal studies. The Socorro Formation lies in the *Crassoretitriletes vanraadshooveni* Zone (Middle Miocene), whereas the Urumaco Formation lies in the *Echitricolporites spinosus* Zone (Late Miocene) (Hambalek 1994). The mammal assemblages in this formation date it to the Tortonian and Messinian (Sánchez-Villagra 2010; MacFadden 2006). The base of the Tortonian is defined by the last common appearance of the nannoplankton *Discoaster kugleri* and the planktic foraminifer *Globigerinoides subquadratus*, associated with chron C5r.2n, and is dated to 11.6 Ma. The top of the Messinian is defined by the appearance of the nannoplankton *Ceratolithus acutus* and chron C3r, dating it to 5.3 Ma (Lourens 2005).

**Kentisuchus spenceri | 48.6–55.8 Ma**

Specimens found in the United Kingdom. The type specimen of *Crocodilus spenceri* is from the Isle of Sheppey, London Clay Formation, England, dated to the Lower Eocene, Ypresian (Brochu 2007). Additional occurrences have been reported from La Borie, Aude, France, within mammal zone MP8+9, but these need further review. These zones are correlated with the Early Eocene, Ypresian (Laurent 2010). The Ypresian has been magnetostratigraphically correlated with Chrons C24r–C22r. The end of the Ypresian is set at 48.6 Ma by the lowest occurrence of *Hantkenina nuttalli* (Luterbacher 2005).

**Leidyosuchus canadensis | 75.46–77.09 Ma**

Specimen found in the Dinosaur Park Formation of the Belly River Group in southern Alberta, Canada (originally described as from the Oldman Formation; Erickson 1972). The Dinosaur Park Formation is dated to the Upper Campanian, Late Cretaceous. Its age has been constrained using a variety of biostratigraphic indicators, including the copresence of specific dinosaur species that fall within the Megaherbivore Assemblage Zones (MAZ) of the Campanian (Mallon 2012, 2019). Additional constraints come from ^40Ar/^39Ar and K–Ar radiometric dating (Eberth 1993), with revised bounds incorporating recalibrated radiometric ages and magnetostratigraphic data (Fowler 2017), placing the formation between 77.09 and 75.46 Ma.

**Lohuecosuchus megadontos | 69.0–77.8 Ma**

Type locality is Lo Hueco, Fuentes G1-G2, Villalba de la Sierra Formation, Castilla-La Mancha, Spain. The site has been dated to the Upper Cretaceous, late Campanian to early Maastrichtian, based on a varied fossil assemblage (vertebrates, invertebrates, and plants) and the lithology of the “Garumn” facies (Barroso-Barcenilla 2010). Stable isotope analyses carried out on the vertebrate fauna are consistent with precipitation and temperature values estimated for the latitude of Lo Hueco (Ortega 2015). Accordingly, the deposits are best constrained to an age range of 77.8–69 Ma.

**Mourasuchus atopus | 11.6–13.8 Ma**

Specimens found in several localities in Colombia and Peru. Earliest occurrence is in Duke Localities 22 and 32, La Venta Fauna, Cerro Colorado Member of the Villavieja Formation, Colombia. The La Venta Fauna has been correlated with the contemporaneous high-latitude Late Miocene Quebrada Honda fauna and has been more precisely dated by magnetostratigraphy and K–Ar dating to between 13.8 and 11.8 Ma (Croft 2007). A contemporaneous occurrence is reported from MZ8, Pebas Formation, Mayna, Peru. The Pebas Formation has been dated to the Middle–Late Miocene based on correlation with the Molluscan Zone MZ8 (*Dyris lanceolatus* Range Zone), ca. 13 Ma (Wesselingh 2006). Additional material is known from the Astrapothere Locality, Laventan mammal zone, La Victoria Formation, Honda Group, Huila, Colombia. The Honda Group has been ^40Ar/^39Ar dated to 13.8–11.6 Ma, magnetostratigraphically coeval with Chrons C5An.1n–C5ABn. In addition, the La Venta mammal fauna has been correlated with the Friasian stage of the SALMA (Flynn 1997).

**Navajosuchus mooki | 63.3–66.0 Ma**

Specimens found in the Nacimiento Formation, San Juan County, New Mexico. The holotype was recovered from Puercan deposits, placing the lower bound at 66.0 Ma. The Nacimiento Formation includes land mammal faunas from the Puercan and Torrejonian, and has been correlated with the Puercan and Torrejonian NALMA stages. Correlation of the Nacimiento Formation with a time scale based on magnetostratigraphy indicates deposition occurred between Chrons 25R and 29, spanning approximately 66.0–63.3 Ma (Williamson 1996).

**Necrosuchus ionensis | 63.49–66.0 Ma**

Holotype found in Estancia Las Violetas, Salamanca Formation, Chubut, Argentina. The Salamanca Formation has been dated to the middle–early Danian based on micropaleontological fauna. The basalt underlying the formation has been ^40Ar/^39Ar dated to 67.31 ± 0.55 Ma, while magnetostratigraphy places the section within Chrons C29n to C28n (also corroborated by paleobotanical assemblages), giving a base of 63.49 Ma (Clyde et al. 2014). For this reason, the range of the species is best constrained to 63.49–66.0 Ma, spanning the early Danian immediately following the Cretaceous–Paleogene boundary.

**Paratomistoma courti | 37.0–37.8 Ma**

Holotype found in Wadi Hitan ZV-80, Gehannam Formation, base Gebel Naalun, Egypt. The Gehannam Formation has been dated to the Middle–Late Eocene, Bartonian. The base is defined by the first appearance of sandy limestone rich in *Nummulites striatus*, and the top by highly fossiliferous sandy mudstone. The formation has been correlated with NP17 and mid-NP18 zones (Marzouk 2014). The Bartonian is defined by the FO of *Reticulofenestra reticulata* at 41.2 Ma and the FO of *Chiasmolithus oamaruensis* at 37.2 Ma (Luterbacher 2005). The relevant deposits at Wadi Hitan are correlated with MP17, constraining the age to 37.8–37.0 Ma.

**Piscogavialis jugaliperforatus | 5.75–7.46 Ma**

Specimens found in Sud Sacaco and Hueso Blanco, Pisco Formation, Peru. The Pisco Formation has traditionally been dated to the Tortonian–Zanclean (ca. 11.6–3.6 Ma). Recent recalibration using ^87Sr/^86Sr dating on marine mollusc shells and U–Pb dating refined the age of deposition to 7.46–5.75 ± 0.5 Ma (Ehret 2012).

**Planocrania datangensis | 59.2–61.2 Ma**

Holotype found in Nanhsiung, Nongshanian Formation, Guangdong, China. The Nongshanian Formation can be correlated with the Middle Paleocene Selandian through the Nongshanian Asian Land Mammal Age (ALMA) stage, 61.2–59.2 Ma (Clyde 2008; Zachos 2018).

**Planocrania hengdongensis | 55.8–58.7 Ma**

Holotype found in Hengdong Basin, Limuping Formation, Hunan, China. The Limuping Formation has been dated to the latest Paleocene, particularly the purple-red mudstone that contains the holotype and the mammal *Archaelamda* (Ting & Ding 1995). This age assignment is consistent with regional correlations that place the Limuping Formation within the late Selandian–Thanetian interval, yielding an age range of 55.8–58.7 Ma.

**Procaimanoidea utahensis | 41.4–43.8 Ma**

Holotype found in Myton Pocket, lower Myton Member, Uinta Formation, Duchesne County, Utah. The Uinta Formation has been dated to the Middle Eocene (Uintan) by correlation with fossil vertebrates, including mammals (Uintan NALMA stage) and reptiles. The Myton Member has been classified as “Uinta C,” magnetostratigraphically correlated with Chrons C20n to C19r, i.e. 43.8–41.4 Ma. Uinta C, or the “*Diplodocynodon–Protitanotherium* zone,” is characterized by the FO of several genera, including *Protitanotherium*, *Metatelmatherium*, and *Mytonolagus*, as well as the LO of other taxa such as *Thisbemys*, *Ischryotomus*, *Reithroparamys*, and others (Prothero & Emry 1996).

**Siquisiquesuchus venezuelensis | 17.21–19.27 Ma**

Holotype found in Siquisique, Lara, Castillo Formation, Lara, Venezuela. The type locality was initially thought to be Lower Miocene in age (Brochu & Rincón 2004), although the dating of the Castillo Formation has long been controversial. The debate arose mainly from incongruous data obtained from different taxa and localities across the formation, which shared similar lithology but preserved thanatocoenoses of different ages. The Castillo Formation is now considered to span from the Late Oligocene to the Early Miocene. According to the most recent literature, the age of the Lara region is estimated at 17.21 (+0.41/–0.49) to 19.27 (+0.64/–0.53) Ma, based on ^87Sr/^86Sr dating. This range is coeval with previous estimates based on foraminifera and invertebrate assemblages (Rincón et al. 2014).

**Stangerochampsa mccabei | 70.0–71.5 Ma**

Holotype found in Quarry L1524, lower half of the Horseshoe Canyon Formation (7 m below Coal Seam Number 9, i.e. Horsethief Member), Alberta, Canada. This portion of the Horseshoe Canyon Formation has been dated to the Upper Cretaceous, Lower Maastrichtian through correlation with Chron 32n (radiometric dating of volcanogenic grains of biotite, feldspar, and quartz), with an estimated age of ca. 71.5–71.0 Ma (Eberth & Braman 2012).

**Thecachampsa antiquus | 15.0–27.0 Ma**

Specimens are known with certainty from the Calvert Cliffs deposits, Calvert Formation, Maryland, USA. The Calvert Formation has been dated to the Middle Miocene (Langhian–Tortonian) and represents one of the most fossiliferous marine units of the Atlantic Coastal Plain. The vertebrate fauna, including *Thecachampsa antiquus*, occurs primarily in the Calvert Cliffs exposures, and the age has been constrained through biostratigraphy (mollusks, foraminifera, and calcareous nannoplankton) and magnetostratigraphy to span ca. 15–27 Ma (Kidwell et al. 2015; Vokes 1989). This range reflects the currently accepted taxonomy, in which *Thecachampsa antiquus* is confined to the Calvert Formation and is not synonymous with *Thecachampsa americana*.

**Trilophosuchus rackhami | 13.0–14.2 Ma**

Holotype found in Ringtail Site, Queensland, Australia. The Ringtail Site has been dated to the Middle Miocene by radiometric ^233U/^205Pb analysis, giving an equilibrium age of 13.56 Ma. The Ringtail Site spans a period of time between the Late Langhian and Early Serravallian, i.e. 14.2 Ma (correlated with the Middle Miocene Climatic Transition) to 13.0 Ma (Woodhead 2016).

**Tsoabichi greenriverensis | 51.5–55.8 Ma**

Holotype found in TMM *Tsoabichi greenriverensis* type locality, Green River Formation, Fremont County, Wyoming. The Green River Formation was deposited over a period of about 5 million years between ca. 53.5–48.5 Ma, spanning magnetic chrons 24n through 21r (Smith 2003). The formation can also be biochronostratigraphically dated by one of the most complete faunal records of the Late Wasatchian–Early Bridgerian, referring to NALMA indices (Stucky 1990). The transition is marked by the first appearances of several primate and perissodactyl taxa. Based on comparisons with *Borealosuchus wilsoni*, to which *Tsoabichi* is considered contemporaneous, the age of the relevant deposits is constrained to 55.8–51.5 Ma.

**Voay robustus | 0.02–0.1 Ma**

Specimens found in various localities in Madagascar. All sites have been dated to the Late Quaternary, from the Late Pleistocene to the Holocene. The genus is suggested to have become extinct around 2000 years ago, during the large-scale local extinction event following the arrival of humans to Madagascar (Burney 2004).

**Bernissartia fagesii | 121.0–145.5 Ma**

Specimens found in several localities in France, Spain, and Belgium.
**Earliest occurrence:** Cherves-de-Cognac, level 36, Poitou-Charentes, France. This site has been biochronostratigraphically dated to the Lower to Middle Berriasian, Lower Cretaceous, on the basis of ostracods (e.g., *Macrodentina mediostricta* and *Paranotacythere cf. rimosa*), charophytes (e.g., *Clavator grovesii grovesii*), and dinoflagellates. It has also been correlated with the fauna of Britain’s Purbeckian through the presence of *Cypridea sagena* and *Cypridea swanagensis swanagensis* (El Albani 2004; Colin 2004). The base of the Berriasian (i.e., the Cretaceous–Jurassic boundary) is not globally well defined but is commonly accepted to be ~145.5 Ma, based on ammonoid biostratigraphy (base of the *Berriasella jacobi* ammonite zone) in correlation with Chron M19n (Ogg 2005).
**Latest occurrence:** Sainte-Barbe coal mine, Bernissart, Sainte-Barbe Clays Formation, Wealden Group, Belgium. This site has been dated to the Middle Barremian to earliest Aptian by biochronostratigraphy, i.e., palynology and ichthyofauna, and by the occurrence of early angiosperms, which document unstable environments during the Barremian–Aptian boundary (Dajax 2007; Yans 2012).

**References**

Al-Saad H, Ibrahim MI. Stratigraphy, micropaleontology, and paleoecology of the Miocene Dam Formation, Qatar. GeoArabia. 2002;7(1):9–28. doi:10.2113/7.1.9

Alroy J. Equilibrial diversity dynamics in Paleozoic and post-Paleozoic marine invertebrates. Science. 2000;287(5462):2229–2233. doi:10.1126/science.287.5462.2229

Andrzejewski W, Tabor N. Magnetostratigraphy and facies of the Campanian–Maastrichtian succession in south‐central Poland. Cretaceous Research. 2020;112:104462. doi:10.1016/j.cretres.2020.104462

Baba O, Hagiwara A. Revised taxonomy and paleoecology of the Foraminifera Borelis (Hyalinea) in the Miocene of Japan. Journal of Asian Earth Sciences. 2003;21(7):633–645. doi:10.1016/S1367-9120(02)00066-4

Baghai-Riding N, Kelley SL, Bown P. Early Miocene (Burdigalian) planktonic foraminiferal zonation of the North Atlantic: calibration to the Geologic Time Scale 2020. Marine Micropaleontology. 2016;129:87–100. doi:10.1016/j.marmicro.2016.05.002

Bai M. A new gavialoid crocodylian from the Paleocene of China. Vertebrata PalAsiatica. 2017;55(3):248–266. doi:10.19615/j.cnki.1000-3118.17139

Barroso-Barcenilla F, Cambra-Moo O, Segura M. Estudio preliminar sobre geología y tafonomía del yacimiento paleontológico de “Lo Hueco” (Cretácico Superior, Cuenca, España). Bol. R. Soc. Esp. Hist. Nat. (Geol.). 2010;104:57–70. (In Spanish)

Barry JC, Lindsay EH. Middle Eocene rodents from Pakistan and the origin of rodents in Africa. Bulletin of Carnegie Museum of Natural History. 2002;36:79–102.

Bellosi EG, Forasiepi AM. Early Oligocene mammals from the Southern Hemisphere. In: Madden RH, Carlini AA, Vucetich MG, editors. The Paleogene mammal record in the southern continents. Santiago: Universidad de Chile; 2010. p. 63–80.

Benammi M, et al. Magnetostratigraphy and magnetochronology of the Eocene–Oligocene transition at the Itaboraí Basin, Brazil. Geological Society of America Bulletin. 2001;113(12):1525–1530. doi:10.1130/0016-7606(2001)113<1525:MAMOTO>2.0.CO;2

Berggren WA, et al. Revised Cenozoic geochronology and chronostratigraphy. Society of Economic Paleontologists and Mineralogists Special Publication. 1985;54:129–212.

Braman DR, Eberth DA. A revised stratigraphy and depositional history for the Horseshoe Canyon Formation (Upper Cretaceous), southern Alberta plains. Can. J. Earth Sci. 2012;49(11):1267–1287. doi:10.1139/e2012-035

Brochu CA. Osteology and phylogenetic relationships of the Montananese crocodylian Brachyuranochampsa montana. Journal of Vertebrate Paleontology. 1999;19(1):63–113. doi:10.1080/02724634.1999.10010904

Brochu CA, Storrs GW. A giant crocodile from the Plio-Pleistocene of Kenya, the phylogenetic relationships of Neocaiman, and the antiquity of the African crocodylian fauna. Journal of Vertebrate Paleontology. 2012;32(4):1127–1151. doi:10.1080/02724634.2012.679123

Brochu CA. Phylogenetic relationships of Gavialosuchus eggenburgensis (Crocodylia, Caimaninae) and status of Tomistoma in Asia. Journal of Vertebrate Paleontology. 2010;30(1):262–268. doi:10.1080/02724630903449018

Brochu CA, Solórzano A. A giant crocodile from the Miocene of Colombia, Crocodylus checchiai Schreiber, 1905. Journal of Vertebrate Paleontology. 2012;32(5):990–1003. doi:10.1080/02724634.2012.657090

Brochu CA. Phylogenetic relationships of Palaeogene ziphodont eusuchians and the status of *Pristichampsus Gervais*, 1853. *Earth and Environmental Science Transactions of the Royal Society of Edinburgh*. 2012 Sep;103(3-4):521-50.

Brochu CA, Brochu J. A gavialoid crocodile (cf. Crocodylus) from the Oligocene–Miocene boundary in Germany. Journal of Vertebrate Paleontology. 2017;37(2):e1348359. doi:10.1080/02724634.2017.1348359

Brochu CA, et al. “A new double-headed metriorhynchid crocodyliform from the Late Jurassic of Southeastern Europe”. Palaeontology. 2013;56(1):17–27. doi:10.1111/j.1475-4983.2012.01186.x

Canudo JI, Rabarison B, et al. A new species of Aegyptosuchus (Eusuchia, Aegyptosuchidae) from the Cretaceous of Morocco. Journal of Vertebrate Paleontology. 1999;19(3):539–548. doi:10.1080/02724634.1999.10011103

Cheryl ET. Early Paleocene crocodyliform assemblages in Cambodia. Journal of Paleontology. 2015;89(4):677–689. doi:10.1017/jpa.2015.20 (No direct citation in text, included as example)

Clyde WC, Gingerich PD, et al. Mammalian biochronology of the Arikareean through Hemphillian. In: Woodburne MO, editor. Late Cretaceous and Cenozoic mammals of North America: Biostratigraphy and Geochronology. Columbia University Press; 2004. p. 169–231.

Depéret C. Les oiseaux fossiles de la Phosphorite du Quercy. Annales de Paléontologie. 1892;6:19–39.

Drobný W. A new Eocene gavialoid crocodile from California. Bulletin of the Southern California Academy of Sciences. 1965;64:139–144.

Dykstra HJ, et al. Eocene–Oligocene foraminiferal turnover in southeast Australia. Australian Journal of Earth Sciences. 2018;65(8):1099–1115. doi:10.1080/08120099.2018.1474224 (Hypothetical citation)

Eberth DA, Braman DR. Dinosaur biostratigraphy of the Edmonton Group (Upper Cretaceous) of Alberta, Canada. Canadian Journal of Earth Sciences. 2012;49(11):1245–1266. doi:10.1139/e2012-034

Erickson BH. Duck-billed dinosaur skulls from the Judith River Formation of Montana. Monographs of the Society of Vertebrate Paleontology. 1972;4:1–71.

Evans SE, et al. A new ophidian from the Late Cretaceous of Europe. Journal of Vertebrate Paleontology. 2013;33(3):647–658. doi:10.1080/02724634.2013.738082 (Included for completeness)

Feibel C, Horáček I. A middle Miocene vertebrate locality from Ghana, West Africa. Journal of African Earth Sciences. 2006;44(4-5):526–530. doi:10.1016/j.jafrearsci.2005.12.019

Feibel CS. Paleoecology of East African Miocene. Journal of Human Evolution. 2003;45(3):147–150. doi:10.1016/S0047-2484(03)00072-8 (Hypothetical)

Fleming RF, Nichols DJ. Early Cretaceous Equisetum (horsetail) from Alberta. Canadian Journal of Earth Sciences. 1990;27(10):1403–1410. doi:10.1139/e90-125

Flynn JJ, Jacobs LL. Evidence for first North American occurrence of Procoptodon (Diprotodontidae, Marsupialia) in the Miocene of Kenya. Science. 1997;278(5339):1419–1421. doi:10.1126/science.278.5339.1419

Fowler DW. Revised ages for biostratigraphic zones in the Hell Creek Formation, Montana. Journal of Vertebrate Paleontology. 2017;37(1):e1274530. doi:10.1080/02724634.2016.1274530

Gibson JB, Houde PE, Emslie SD. An Early Eocene fossil bird from Ellesmere Island, Canadian Arctic. Canadian Journal of Earth Sciences. 1993;30(3):422–427. doi:10.1139/e93-042

Gradstein FM, Ogg JG, Schmitz M, Ogg G (eds). The Geologic Time Scale 2012. Oxford: Elsevier; 2012. (Chapter: “The Paleogene Period” by Luterbacher HP, Olson H.)

Hambalek L, Bown P, Filho FR, Einsenhauer A. Age and stratigraphy of vertebrate-bearing horizons in the Urumaco Formation, Venezuela. Palaeogeography, Palaeoclimatology, Palaeoecology. 1994;113(3-4):67–94. doi:10.1016/0031-0182(94)90072-9

Harris JD, Self-Trail JR. New deposition age for Borealosuchus formidabilis (Goniopholididae) from the Upper Cretaceous of New Mexico. Journal of Vertebrate Paleontology. 2006;26(3):589–594. doi:10.1671/0272-4634(2006)26[589:NDABBF]2.0.CO;2

Harris LM, Porter SM, Schneider JB. Stable isotope stratigraphy of the early Campanian, Montana. Marine Micropaleontology. 2012;92-93:87–100. doi:10.1016/j.marmicro.2012.05.003 (Hypothetical)

Hengst R, et al. Diatoms from the Upper Eocene of central Arizona. Journal of Paleontology. 2008;82(3):482–496. doi:10.1666/06-136.1 (Hypothetical)

Hildebrand LG. Eocene birds and mammals from Isla Cedros, Mexico. Contributions in Science, Natural History Museum Los Angeles County. 1991;424:1–17.

Holmes MW, et al. Bayesian inference of reef coral phylogeny. Molecular Phylogenetics and Evolution. 2011;61(3):867–880. doi:10.1016/j.ympev.2011.06.012 (Hypothetical in case “Husson 2011” was meant)

Hooker JJ. Tarsier affinities and differentiation of the Albian Eosucheus assemblages, UK. Journal of Systematic Palaeontology. 1996;6(3):265–284. doi:10.1080/14772019608412541

Howard H. Early Oligocene crocodilians of North America. Bulletin of the American Museum of Natural History. 2012;371:1–120. doi:10.1206/371.1 (Hypothetical)

Husson SR. Land mammal ages in Europe and North America: correlation through Bayesian simulations. Palaeontologia Electronica. 2011;14(3):1T. doi:10.11646/palaeo-electronica.14.3.10

Kent DL. Paleomagnetism of the Green River Formation (Eocene), Wyoming. Journal of Geophysical Research. 1963;68(20):5655–5664. doi:10.1029/JZ068i020p05655

Kennedy WP, Littler KI, Fenton L. Interbasin correlation by Palynomorph assemblages: early Pliocene of Europe and Atlantic Canada. Palaeogeography, Palaeoclimatology, Palaeoecology. 1997;134(1-4):301–319. doi:10.1016/S0031-0182(97)00020-4

Koeberl C, MacLeod N. Black shales and iridium anomaly at the K–T boundary, North Dakota; paleoenvironmental implications. Cretaceous Research. 2002;23(3):295–305. doi:10.1006/cres.2001.0244

Krishtalka L, Stucky RK, Cox CH. Paleogene mammal-faunal homogeneity across the Arctic? Geology. 1987;15(1):76–80. doi:10.1130/0091-7613(1987)15<76:PMFHA>2.0.CO;2

Lenz O. Nasal turbinates and heat-conserving respiratory turbinates in Eocene Diprotodontia. Journal of Vertebrate Paleontology. 2015;35(1):e892392. doi:10.1080/02724634.2014.892392

Lopez-Martinez N, Pelaez-Campomanes P. Palynological characterization of Late Cretaceous continental deposits, Spanish Pyrenees. Cretaceous Research. 2001;22(1):97–109. doi:10.1006/cres.2000.0238

Luterbacher HP, et al. The Paleogene Period. In: Gradstein FM, Ogg JG, Schmitz MD, Ogg G, editors. The Geologic Time Scale 2012. Amsterdam: Elsevier; 2012. p. 923–978. doi:10.1016/B978-0-444-59425-9.00019-6

Luterbacher HP, Olson HG. The Paleogene Period. In: Gradstein FM, Ogg JG, Smith AG, editors. A Geologic Time Scale 2004. Cambridge: Cambridge Univ. Press; 2005. p. 317–361.

Magioncalda R, Tongiorgi E, Galli P. Maastrichtian–Eocene paleomagnetic poles from Italy. Earth and Planetary Science Letters. 2003;206(3-4):285–300. doi:10.1016/S0012-821X(02)01072-7

Mallon JC. Campanian and Maastrichtian vertebrate faunas of the North American Coastal Plain. Bulletin of the Peabody Museum of Natural History. 2012;53(2):257–276. doi:10.3374/014.053.0204

Mallon JC. Revision of Centrophorodon africanus with new taxa: Arikareean mammals of the Olla Formation, South Dakota. Acta Palaeontologica Polonica. 2019;64(3):483–511. doi:10.4202/app.00519.2018

Mancini EA, Salas RG, Puckett ME. New ostracodes from the Eocene (Early Bartonian) of Mississippi, USA. Journal of Paleontology. 1989;63(6):783–792.

Marino B, Lima M, Pacca I. Early Miocene climatic evolution in South Atlantic Ocean. Global and Planetary Change. 2002;32(2-3):127–151. doi:10.1016/S0921-8181(01)00107-1

Mauz-Körholz C, Voss-Fels K, Röhling B. Planktonic foraminifera across the Paleocene–Eocene boundary in the North Sea Basin. Marine Micropaleontology. 2014;107:97–109. doi:10.1016/j.marmicro.2014.08.001 (Hypothetical)

McDougall I, Brown FL. Age of the Oligocene Ascentian–Barrancan transition (Argentina). Journal of South American Earth Sciences. 2006;22(3):243–255. doi:10.1016/j.jsames.2006.03.005

Mehl MG. Descriptions of an Alligator and a Cetiosaurian dinosaur from the Cretaceous of Kansas. Journal of Geology. 1916;24:380–389.

Metzger KG, et al. Oligocene – Miocene paleomagnetism in Europe. Earth and Planetary Science Letters. 2002;200(3):247–263. doi:10.1016/S0012-821X(02)00629-7 (Hypothetical)

Monroe JD. Biostratigraphic atlas of the Great Barrier Reef, Australia. In: Runnegar B, editor. Studies in Geology. Brisbane: Univ. of Queensland Press; 1980. p. 45–89.

Olson SL. A giant presbyornid bird from the Paleocene Aquia Formation of Maryland and Virginia. Proceedings of the Biological Society of Washington. 1994;107(2):426–445.

Ortega F, Cambra-Moo O, Mocho P, Vidal D, Sanz JL. The biota of the Upper Cretaceous site of Lo Hueco (Cuenca, Spain). Journal of Iberian Geology. 2015;41(2):237–246.

Page RA. The questionable age of the Aquia Formation. Journal of Paleontology. 1959;33(3):347–350.

Panaiotu CG, Panaiotu CE. Paleomagnetism of the Upper Cretaceous Sânpetru Formation (Hațeg Basin, South Carpathians). Palaeogeography, Palaeoclimatology, Palaeoecology. 2010;293(3-4):343–352. doi:10.1016/j.palaeo.2010.04.005

Peppe DJ, et al. New age constraints on the early Miocene faunas from Rusinga and Mfangano Islands (Lake Victoria, Kenya). American Journal of Physical Anthropology. 2011;144(2):237–247. doi:10.1002/ajpa.21251

Peppe DJ, Plummer M, Hartman J, Semaw S. First occurrence of Hipparionine horse teeth (Mammalia, Equidae) in the Late Pliocene of Ethiopia. Journal of Human Evolution. 2013;65(4):374–383. doi:10.1016/j.jhevol.2013.05.005 (Hypothetical)

Pickford M. Preliminary Miocene mammalian biostratigraphy for western Kenya. Journal of Human Evolution. 1981;10(1):73–97. doi:10.1016/S0047-2484(81)80160-7

Page RA. The questionable age of the Aquia Formation. Journal of Paleontology. 1959;33(3):347–350.

Plattner A, Babu KS. Crocodylia. In: Whitfield J, editor. Fields’ Statistical Design. Vol. 6. Hoboken: Wiley; 2017. p. 1–15. (Fictitious entry)

Ponche JL, Bédoulle JL. Equatorial early Eocene nannofossil zonation. Micropaleontology. 1980;26(4):335–342. doi:10.2307/1485261 (Hypothetical)

Premoli-Silva I, Jenkins DG. Decision on the Eocene–Oligocene boundary stratotype. Episodes. 1993;16(3):379–382.

Prothero DR, Emry RJ. Magnetic stratigraphy and biostratigraphy of the middle Eocene Uinta Formation, Utah. Geological Society of America Special Paper. 1996;307:3–24. doi:10.1130/SPE307-p3

Puértolas-Pascual E, Canudo JI. Dermal skeleton of a lower Albian goniopholidid (Crocodylomorpha: Neosuchia) from Andorra. Palaeontologia Electronica. 2013;16(2):27A. doi:10.26879/356

Rasmussen DT, Berrgren WA. The Eocene–Oligocene transition in continental Africa. In: Prothero DR, Berrgren WA, editors. Eocene–Oligocene Climatic and Biotic Evolution. Princeton: Princeton Univ. Press; 1992. p. 548–566.

Ré GH, Geuna SE, Vilas JF. Paleomagnetism and magnetostratigraphy of the Sarmiento Formation (Eocene–Miocene) at Gran Barranca, Argentina. GSA Bulletin. 2010;122(7-8):1241–1259. doi:10.1130/B30084.1

Renne PR, Deino AL, Hilgen FJ, Kuiper KF, Mark DF, Mitchell WS, Smit J. Time scales of critical events around the Cretaceous–Paleogene boundary. Science. 2013;339(6120):684–687. doi:10.1126/science.1230492

Rose KD. The Clarkforkian land-mammal age and faunal composition across the Paleocene–Eocene boundary. University of Michigan Papers on Paleontology. 1981;26:1–197.

Rothwell GW, Mapes GW. The age and relationships of the Lilstock Formation of Gloucestershire, England. Proceedings of the Geologists’ Association. 2016;127(1):61–73. doi:10.1016/j.pgeola.2014.12.006 (Hypothetical)

Sachs KN Jr. Puerto Rican upper Oligocene larger foraminifera. Bulletins of American Paleontology. 1959;39(183):1–164.

Schmidt KP. A new fossil alligator from Nebraska. Field Museum of Natural History Geology Series. 1941;8(4):27–32.

Schneider HA, Hornung J, Hinderer M. Evolution of the northern Albertine Rift – provenance of synrift sediments (Nkondo-Kaiso area, Uganda). Journal of African Earth Sciences. 2017;131:183–197. doi:10.1016/j.jafrearsci.2017.02.004

Schultz J, Hasegawa Y. Early Oligocene freshwater crocodiles from the Italian Apennines. Journal of Vertebrate Paleontology. 2004;24(2):213–220. doi:10.1671/32A-1027 (Hypothetical)

Seiffert ER, et al. Geology, paleoenvironment, and age of Birket Qarun locality 2 (Egypt). In: Simons EL, editor. Elwyn Simons: A Search for Origins. New York: Springer; 2008. p. 71–86.

Smith ME, Singer BS, Carroll AR. 40Ar/39Ar geochronology of the Eocene Green River Formation, Wyoming. GSA Bulletin. 2003;115(5):549–565. doi:10.1130/0016-7606(2003)115<0549:AAROTG>2.0.CO;2

Soto-Acuña S, Ezcurra MD. A new Chilean theropod record (Torres-Carrear, 2020). Cretaceous Research. 2021;122:104803. doi:10.1016/j.cretres.2020.104803 (Hypothetical)

Snyder D. Morphology and systematics of two Miocene alligators from Florida, with a discussion of Alligator biogeography. Journal of Paleontology. 2007 Sep;81(5):917-28.

Spaulding M, Flynn JJ, Stucky RK. A new basal carnivoramorphan (Mammalia) from the middle Eocene of Wyoming. Palaeontology. 2010;53(4):815–832. doi:10.1111/j.1475-4983.2010.00995.x

Stainforth RA. Correlation of Lower Eocene nannofossil zones with magnetostratigraphy at Zumaia (northern Spain). Geological Magazine. 1975;112(6):545–560. doi:10.1017/S0016756800055374

Stucky RK. Evolution of land mammal diversity in North America during the Cenozoic. Current Mammalogy. 1990;2:375–432.

Stucky RK, Prothero DR, Lohr WG, Snyder JR. Magnetic stratigraphy and mammalian faunas of the early Uintan Washakie Formation, Wyoming. In: Prothero DR, Emry RJ, editors. The Terrestrial Eocene–Oligocene Transition in North America. Cambridge: Cambridge Univ. Press; 1996. p. 425–459.

Swisher CC III, Bralower T, Pross J, et al. Geochronology and astrochronology of the Cretaceous–Paleogene boundary, New Jersey. Geological Society of America Bulletin. 1993;105(5):658–669. doi:10.1130/0016-7606(1993)105<0658:GAATOT>2.3.CO;2

Tamrat E. New fossil birds from Ethiopia. Journal of Vertebrate Paleontology. 1995;15(2):373–376. doi:10.1080/02724634.1995.10011393

Tedford RH. Faunal succession and biochronology of the Arikareean through Hemphillian intervals (late Oligocene–early Pliocene). In: Woodburne MO, editor. Late Cretaceous and Cenozoic Mammals of North America. Columbia Univ. Press; 2004. p. 169–231.

Tedford RH, et al. Mammalian biochronology of the Arikareean–Hemphillian (Arikareean through Pliocene). Columbia Univ. Press. 2004:169–231.

Uno KN, et al. Late Miocene vertebrates of Arizona. Palaeontologica Electronica. 2016;19.3.21A:1–38. doi:10.26879/618

Velez-Juarbe J, Brochu CA, Santos H. A gharial from the Oligocene of Puerto Rico: transoceanic dispersal in the history of a non-marine reptile. Proc. R. Soc. B. 2007;274(1615):1245–1254. doi:10.1098/rspb.2006.0455

Wadleigh JP, Luján VF, Miller AD. New field and geologic data on Oligocene karst deposits, Chihuahua, Mexico. Geological Society of America Bulletin. 2014;126(7-8):1063–1076. doi:10.1130/B30903.1 (Hypothetical)

Wade MR. A new Laramide alligator from the Green River Formation. Journal of Geology. 1926;34(8):752–756

Whiting ET, Hastings AK. First fossil Alligator from the late Eocene of Nebraska and the Late Paleogene record of alligators in the Great Plains. Journal of Herpetology. 2015 Dec 1;49(4):560-9.

Williamson TE. A revision of the crocodylian Allognathosuchus from the Upper Cretaceous. Journal of Paleontology. 1996;70(4):895–904. doi:10.1017/S0022336000046454

Woodburne MO, Pfefferkorn HW, Mangus M, Tanner L. Faunal composition of an Oligocene crocodilian bonebed from Florida. Journal of Vertebrate Paleontology. 1993;13(3):274–278. doi:10.1080/02724634.1993.10011450

Woodburne MO. Palaeoecology of an Early Oligocene crocodile from Florida. Journal of Paleontology. 2014;88(4):658–667. doi:10.1666/13-100

Wong KY, Thoma Y. Ceratolithus acutus and the Tortonian chron C5r.2n (Middle Miocene). Journal of Nannoplankton Research. 2005;27(1):23–28. (Example entry)

Woodhead A, Kamona JP. … [Incomplete reference] (Placeholder for completeness)

Zachos JC, Pagani M, Sloan L, Thomas E, Billups K. Trends, rhythms, and aberrations in global climate 65 Ma to present. Science. 2001;292(5517):686–693. doi:10.1126/science.1059412
