# Supplementary material for: Competing methods of divergence time estimation recover diverse evolutionary timescales
Source: Proc Biol Sci. 2026 Oct 7;293(2080):20260635. doi: 10.1098/rspb.2026.0635 (PMC13641909; doi:10.1098/rspb.2026.0635)
Supplement: Supplementary file 1 [file rspb.2026.0635.zip › rspb.2026.0635/rspb20260635_si_001.zip!/supp/2.1_node_calibrations.docx]

**Crocodilia Node Calibrations**

**Crocodile-Alligator split | 95-74.997 Ma**

**Fossil taxon and Specimen:** *Leidyosuchus canadensis* (AMNH 5342; holotype) from the Dinosaur Park Formation (below the Lethbridge Coal Member), Belly River Group, Western Canadian Sedimentary Basin, southern Alberta, Canada (Lambe, 1907)**.**

**Phylogenetic justification:** Following Brochu (1997), Wu et al. (2001) demonstrated that *L. canadensis* can be distinctively diagnosed as the sole representative of *Leidyosuchus*, based on more than 10 derived cranial and mandibular features. The status of *L. canadensis* as the basalmost alligatoroid is corroborated by additional derived character states, including a broad anterolateral border of the suborbital fenestra, a broad occlusion surface of the splenial medial to the posterior four dentary teeth, an anterolateral margin of the suborbital fenestra that is shorter than the posterolateral margin, a retroarticular process which does not extend posterodorsally beyond the edge of the articular fossa, and a splenial which is extensively involved in the formation of the dorsomedial wall of the posterior 11 and 12 alveoli. *Leidyosuchus*’ placement within basalmost Alligatoroidea has been corroborated by a recent tip dating study by Lee & Yates (2018) combining morphological, molecular, and stratigraphic data.

**Minimum Age justification:** All specimens of *Leidyosuchus canadensis* are derived from below the Lethbridge Coal Member within the Dinosaur Park Formation (Wu et al. 2001). The Dinosaur Park Formation has been dated using CA‑ID‑TIMS U–Pb by Ramezani et al. (2022) who constrain the minimum age of the Dinosaur Park Formation below the Lethbridge Coal Member to at least 75.017 Ma ± 0.02 Myr. This allows for a minimum constraint of 74.997 Ma.

**Maximum age justification:** Our soft maximum for the root of Crocodylia is based on the recently described taxa *Portugalosuchus azenhae*. Originally inferred to represent the oldest known crocodylian based on morphology alone (Mateus et al. 2019), a plethora of phylogenetic and biogeographical evidence, most notably the fact that this resolution would imply substantial ghost lineages, has revealed *Portugalosuchus* as most likely representing a non-crocodylian eusuchian (Darlim et al. 2022). Subsequently, the occurrence of *Portugalosuchus* at 95 Ma represents a time when the ecologic, geologic, and taphonomic conditions were suitable for the fossilisation of basal crocodylians morphologically comparable to *Portugalosuchus* (such as *Brachychampsa sealeyi*), but that predates their first appearance in the stratigraphic record by around 10 Myr. Following the best practises of Parham et al. 2012, this horizon therefore represents a suitable conservative soft maximum bound for the origin of crown-group Crocodylia, with the time of divergence likely falling somewhere within this interval.

**Additional discussion:** Traditionally, crocodiles and alligators were grouped together in the clade Brevirostres – however, recent molecular studies have rejected this, instead classifying extant alligators and caimans together in the crown group Alligatoridae, the stem group of which (including more basal extinct alligatorid ancestors), is referred to as Alligatoroidea (Harshman et al. 2003, Gatesy and Amato 2008, Erickson et al. 2012, Hekkala et al. 2021).

**Alligator-Caiman split | 83.4-62.53 Ma**

**Fossil taxon and Specimen:** *Necrosuchus ionensis* (AMNH 3219; holotype) from the Salamanca Formation, Rio Chico Group, Golfo San Jorge Basin, Patagonia, Argentina (Simpson, 1937).

**Phylogenetic justification:** *Ne. ionensis* is robustly placed among Caimaninae based on a number of key synapomorphies, including a splenial excluded from the mandibular symphysis with a projection dorsal to the Meckelian groove, an angular-surangular suture contacting the ventral margin of the external mandibular fenestra, a dentary symphysis extending back to a level just behind the fourth dentary alveolus and the presence in *Ne. ionensis* of a slender process of the exoccipital ventral to the basioccipital tubera (Walter et al. 2022).

**Minimum age justification:** The top of the Salamanca Formation falls below the C27r-C27n boundary (Clyde et al. 2014) which is dated to 62.53 Ma in Speijer et al. (2020).

**Maximum age justification:** We set a soft maximum time of divergence for this split to 83.4 Ma based on the occurrence data of *Brachychampsa sealeyi*, the oldest fossil crocodylian ever considered an alligatorid (although this conclusion is the subject of conjecture: see additional discussion). Specimens of *Brachychampsa* derive from the Menefee Formation, San Juan Basin, New Mexico, USA, with the locality having been dated using U-PB estimation between 81-75 Ma (Dickinson and Gehrels, 2009). Since, according to best practise, soft maxima should be set to an older age than the oldest possible fossil record of the taxa in question (Parham et al. 2012), and *Brachychampsa* has been recovered in the past as belonging to Caimaninae (Bona et al. 2018; Stocker et al. 2021), we set our soft maximum at 83.4 Ma, at the base of the Campanian. This justification is largely based on Walter et al. 2022.

**Additional discussion:** Calibrating the split between Alligatorinae and Caimaninae is complicated by the putative affinity of Late Cretaceous basal alligatorids *Brachychampsa*, *Stangerochampsa*, and *Albertochampsa* with caimans, which would push back the this split by approximately 10 million years. However, evidence for this relationship is based on a limited number of derived characters from poorly preserved specimens, and there is insufficient evidence to conclusively diagnose the existence of Cretaceous caimanines. Following best practice, it would be more appropriate to select one of the oldest unambiguous caimanines (or alligatorines) to calibrate this split, with the caimanines *Eocaiman paleocenicus*, *Necrosuchus ionensis*, *Notocaiman stromeri* and *Protocaiman peligrensis*, as well as the alligatorine *Navajosuchus mooki* representing the most applicable candidates. Among these, a comparative dearth of posterior cranial material in *Eo. Paleocenicus*, *No. stromeri*, and *Na. mooki* has resulted in their being less robustly placed among Caimaninae and Alligatorinae, respectively – this indicates they are unstable taxon to be used as fossil constraints for this particular split, and lends credence to *Ne. ionensis* providing a hard minimum fossil age (Walter et al, 2022).

**Crocodile-Osteolaemine split | 17 Ma (minimum)**

**Fossil taxon and Specimen:** *Brochuchus pigotti* (NHMUK PV R7729; holotype) from the Fossil Bed Member of the Hiwegi Formation at Shackleton Gully, Rusinga Island, Kenya (Tchernov and Van Couvering, 1978).

**Phylogenetic justification:** *Brochuchus pigotti* was assigned to osteolaemine crocodylids by Conrad et al. (2013), based on four unambiguous synapomorphies: an ilium with a broad supracetabular crest, prominent preorbital ridges, a pterygoid surface lateral and anterior to the internal choana pushed inward anterolaterally to the choanal aperture, and a squamosal extending ventrolaterally to the lateral extent of the paroccipital process. Additional phylogenetic analyses utilizing molecular, stratigraphical, and morphological data also recovers this assemblage of Osteolaeminae (Hekkala et al., 2021; Lee and Yates, 2022).

**Minimum age justification:** Traditionally, the Hiwegi Formation was interpreted by Drake (1988) as undergoing rapid deposition in an interval of <0.5my, with the majority of fossils dating to ca. 17.8 Ma using K-Ar geochronology. However, leaching and potassium loss due to diagenesis coupled with the inheritance of Precambrian sediments as a result of the Kisingiri eruption have recently called these dates into question, with recent Ar/Ar dating and magnetostratigraphy suggesting a period of deposition of ca. 20-17 Ma, during the Burdigalian (Peppe 2017; Michel et al., 2020; Baumgartner and Peppe, 2021). On this we can establish a 17 Ma minimum age constraint on the Crocodile-Osteolaemine divergence.

**Crown-group *Alligator* split | 14.8 Ma (minimum)**

**Fossil taxon and specimen:** *Alligator thomsoni* (AMNH 1736; holotype) from the Subcycle 1 Member of the Olcott Formation at Trojan Quarry, Sioux County, Nebraska, USA (Mook, 1923).

**Phylogenetic justification:** Crown-group alligatorines contain extant alligators and their closest extinct relatives, including the comparatively basal *Alligator thomsoni*. The latter is considered a closer relative of the extant *Alligator mississippiensis* than its Chinese counterpart, *Alligator sinensis*, based on sutural similarities and the morphology of its prefrontal pillar, suggesting A. Thomsoni lies within the crown-genus Alligator. This is corroborated by additional phylogenetic analysis utilising a combination of molecular, stratigraphical, and morphological data, supporting the notion that A. Thomsoni represents the most suitable calibration for the node characterising the divergence of extant alligators (Lee and Yates, 2018).

**Minimum age justification:** The Olcott Formation has been dated using biochronostratigraphy to the Barstovian terrestrial horizon, which is a North American middle-Miocene faunal stage as per the North American Land Mammal Ages (NALMA) chronology. Within this horizon, the Trojan Quarry locality has been assigned to Biochron Ba1, which corresponds to 16-14.8 Ma (Skinner et al. 1967; Tedford et al. 2004; Whiting and Head, 2020). Subsequently, we can assign a minimum age of divergence for this clade as 14.8 Ma.

**Crown-group Caimaninae split | 18.64 Ma (minimum)**

**Fossil taxon and specimen:** *Cenentariosuchus gilmorei* (UF 262800; holotype) from the Cucaracha Formation at Hodges Hill, Panama (Hastings et al., 2013).

**Phylogenetic justification:** *Cenentariosuchus gilmorei* has been recovered within multiple different rearrangements of Caimaninae in phylogenetic analyses - but, crucially, always within the crown-group (Hastings et al., 2016; Bona et al., 2018; Cidade et al., 2020; Cossette, 2020).

**Minimum age justification:** The top of the Cucaracha Formation falls below the C5En-C5Er boundary (MacFadden et al., 2014), which is dated to 18.64 Ma in Raffi et al. (2020).

**Additional discussion:** While multiple Late Cretaceous-Eocene alligatorid specimens (inc. *Tsoabichi greenriverensis*, *Necrosuchus ionensis*, *Bottosaurus harlani*, and *Orthogenysuchus olseni*) have been recovered within crown-group Caimaninae in various phylogenetic analyses (Brochu, 2011; Bona et al. 2018; Godoy et al. 2021), the unstable nature of this resolution - as well as recent taxonomic revisions of *Or. olseni* based on reinterpretation of key morphological features - makes their candidacy for a prospective calibration point inappropriate (Salas-Gismondi et al. 2015; Cidade et al. 2020; Cossette, 2020). Other genera, including *Mourasuchus* and *Purussaurus*, have also been occasionally recovered in the crown-group (Pinheiro et al. 2013; Fortier et al., 2014), but more fossil evidence is needed before we can reliably diagnose this phylogenetic resolution and consider them as putative calibration species (Walter et al. 2022).

**Crocodile-Tomistomine split | 53.9 Ma (minimum)**

**Fossil taxon and specimen:** *Kentisuchus spenceri* (BMNH 19633; holotype) from the London Clay Formation at the Isle of Sheppey, Kent, England (Buckland, 1836).

**Phylogenetic justification:** Phylogenetic analysis supports a basalmost placement of *Kentisuchus* among tomistomines, less closely related to *Tomistoma* than contemporaneous taxa such as *Dollosuchoides densmorei* and *Gavialosuchus americanus* (Brochu, 2007). This resolution is based on the lacrimal margin being disrupted by a posterior process of the maxilla, a structure diagnostic for Tomistominae – within this classification, *Kentisuchus’* is recognised as the most basal tomistomine primarily on the basis of the posterior premaxillary process being much shorter than more derived specimens (Jouve, 2016). This conclusion was supported by a recent tip-dating study by Lee and Yates (2018) combining morphological, molecular, and fossil age data.

**Minimum age justification:** The London Clay Formation Division B at Sheppey, Kent coincides with the start of Chron C24n.3n (Ali and Jolley, 1996), which is dated to 53.9 Ma, during the Ypresian of the Early Eocene (Speijer et al. 2020).

**Additional discussion:** Calibrating this split has the potential to be complicated by the unstable position of *Gavialis gangeticus* within the crocodylian phylogeny. This is because, formally, Crocodyloidea (a clade subtended by the node in question) includes *Crocodylus niloticus* and any taxa closer to it than *Alligator mississippiensis* or *Gavialis*. Consequently, if *Gavialis* is basal to all other extant crocodylians, as some morphological studies suggest (Brochu, 1997), then the Maastrichtian stem longirostrian *Prodiplocynodon* is implicated as a basal crocodyloid, pushing back the minimum age of divergence for the calibrated node by around 10 Mya. However, analyses of mitochondrial and nuclear gene sequences of *Gavialis* and *Tomistoma schlegelii,* as well as recent phylogenetic tip-dating studies, support the view that they are sister taxa, drawing *Gavialis* closer to *Crocodylus* and keeping the minimum age of divergence for this split in the Early Eocene (Gatesy and Amato, 1992; Harshman et al. 2003; Willis et al. 2007; Lee and Yates, 2018). If the mekosuchines – which are also recovered from strata dating to the Ypresian – are closely related to *Crocodylus*, as many analyses suggest (Rio and Mannion, 2021), then the oldest crocodyloids and gavialoids are approximately the same age, lending credence to the appropriateness of our minimum age justification.

**Reference List**

Ali JR, Jolley DW. Chronostratigraphic framework for the Thanetian and lower Ypresian deposits of southern England. Geol Soc London Spec Publ. 1996;101(1):129–144.

Baumgartner L, Peppe DJ. New chronostratigraphic constraints on the Hiwegi Formation (early Miocene) from Rusinga Island, Kenya. J Afr Earth Sci. 2021;180:104209.

Bona P, Ezcurra MD, Barrios F, Fernández Blanco MV. A new Palaeocene crocodylian from southern Argentina sheds light on the early history of caimanines. Proc Biol Sci. 2018;285(1885):20180843.

Brochu CA. Phylogenetic systematics and taxonomy of Crocodylia. [Unpublished PhD thesis]. University of Texas at Austin; 1997. 467 p.

Brochu CA. Morphology, relationships, and biogeographical significance of an extinct horned crocodile (Crocodylia, Crocodylidae) from the Quaternary of Madagascar. Zool J Linn Soc. 2007;150(4):835–863.

Brochu CA. Phylogenetic relationships of Necrosuchus ionensis Simpson, 1937 and the early history of caimanines. Zool J Linn Soc. 2011;163(Suppl 1):228–256.

Buckland W. Geology and Mineralogy considered with reference to Natural Theology. London: John Murray; 1836.

Cidade GM, Fortier DC, Hsiou AS. Taxonomic and phylogenetic review of Necrosuchus ionensis (Alligatoroidea: Caimaninae) and the early evolution and radiation of caimanines. Zool J Linn Soc. 2020;189(2):657–669.

Clyde WC, Wilf P, Iglesias A, Slingerland RL, Bijl PK, Bralower TJ, et al. New age constraints for the Salamanca Formation and lower Río Chico Group in the western San Jorge Basin, Patagonia, Argentina: implications for Cretaceous–Paleogene extinction recovery and land mammal age correlations. GSA Bull. 2014;126(3–4):289–306.

Conrad JL, Rabi M, Weems RE, Norell MA. A new osteolaemine crocodylid from the Miocene of Kenya. Am Mus Novit. 2013;3769:1–21.

Cossette L, Brochu CA. A systematic review of the giant alligatoroid Deinosuchus from the Campanian of North America and its implications for the relationships at the root of Crocodylia. J Vert Paleo. 2020;40(6):e1767638.

Darlim G, Lee MSY, Walter J, Rabi M. The impact of molecular data on the phylogenetic position of the putative oldest crown crocodilian and the age of the clade. Biol Lett. 2022;18(2):20210603.

Dickinson WR, Gehrels GE. U-Pb ages of detrital zircons in Jurassic eolian and associated sandstones of the Colorado Plateau: evidence for transcontinental dispersal and intraregional recycling of sediment. GSA Bull. 2009;121(3–4):408–433.

Erickson GM, Gignac PM, Steppan SJ, Lappin AK, Vliet KA, Brueggen JD, et al. Insights into the ecology and evolutionary success of crocodilians revealed through bite-force and tooth-pressure experimentation. PLoS One. 2012;7(3):e31781.

Fortier DC, Souza-Filho JP, Guilherme E, Maciente AAR, Schultz CL. A new specimen of Caiman brevirostris (Crocodylia, Alligatoridae) from the late Miocene of southwestern Amazonia, Brazil. J Vert Paleo. 2014;34(4):820–834.

Gatesy J, Amato G. The rapid accumulation of consistent molecular support for intergeneric crocodylian relationships. Mol Phylogenet Evol. 2008;48(3):1232–1237.

Gatesy J, Amato GD. Sequence similarity of 12S ribosomal segment of mitochondrial DNAs of gharial and false gharial. Copeia. 1992;1992(2):241–243.

Godoy PL, Cidade GM, Montefeltro FC, Langer MC, Norell MA. Redescription and phylogenetic affinities of the caimanine Eocaiman cavernensis (Alligatoridae, Crocodylia) from the Paleocene of Argentina. Palaeontogr Abt A. 2021;325(4–6):95–119.

Hastings AK, Bloch JI, Jaramillo CA, Rincon AF, MacFadden BJ. Systematics and biogeography of crocodylians from the Miocene of Panama. J Vert Paleo. 2013;33(2):239–263.

Hastings AK. Character evolution and the origin of Caimaninae (Crocodylia) in the New World tropics: new evidence from the Miocene of Panama and Venezuela. J Paleontol. 2016;90(2):317–332.

Hekkala ER, Gatesy J, Narechania A, Meredith RW, Aardema ML, Montanari S, et al. Paleogenomics illuminates the evolutionary history of the extinct Holocene “horned” crocodile of Madagascar, Voay robustus. Commun Biol. 2021;4:505.

Jouve S. A new basal tomistomine (Crocodylia, Crocodyloidea) from Issel (Middle Eocene; France): palaeobiogeography of basal tomistomines and palaeogeographic consequences. Zool J Linn Soc. 2016;177(1):165–182.

Lambe LM. On a new crocodilian genus and species from the Judith River Formation of Alberta. Proc Trans R Soc Can. 1907;1(4):219–235.

Lee MSY, Yates AM. Tip-dating and homoplasy: reconciling the shallow molecular divergences of modern gharials with their long fossil record. Proc Biol Sci. 2018;285(1881):20181071.

Mateus O, Puértolas Pascual E, Callapez PM. A new eusuchian crocodylomorph from the Cenomanian (Late Cretaceous) of Portugal reveals novel implications on the origin of Crocodylia. Zool J Linn Soc. 2019;186(2):501–528.

Michel LA, Peppe DJ, Lutz AJ, McNulty KP, McBrearty S, Plummer TW, et al. Stratigraphy and age of the early Miocene Hiwegi Formation, Rusinga Island, Kenya. J Hum Evol. 2020;140:102369.

Mook CC. A new species of Alligator from the Miocene of Nebraska. Am Mus Novit. 1923;(77):1–12.

Parham JF, Donoghue PCJ, Bell CJ, Calway TD, Head JJ, Holroyd PA, et al. Best practices for justifying fossil calibrations. Syst Biol. 2012;61(2):346–359.

Peppe DJ, Michel LA, Lutz AJ, McNulty KP, McBrearty S, Plummer TW, et al. Magnetostratigraphy of the early Miocene Hiwegi Formation, Rusinga Island, Kenya. J Afr Earth Sci. 2017;129:36–54.

Pinheiro AEP, Fortier DC, Pol D, Campos DA, Bergqvist LP. A new Eocaiman (Alligatoridae, Crocodylia) from the Itaboraí Basin, Paleogene of Rio de Janeiro, Brazil. Hist Biol. 2013;25(3):327–337.

Ramezani J, Beveridge TL, Rogers RR, Eberth DA, Bakriwei F, Al-Khuzai MH, et al. Calibrating the zenith of dinosaur diversity in the Campanian of the Western Interior Basin by CA–ID–TIMS U–Pb geochronology. Sci Rep. 2022;12:16026.

Rio JM, Mannion PD. Phylogenetic analysis of a new morphological dataset elucidates the evolutionary history of Crocodylia and character changes at the origin of Crocodyliformes. PeerJ. 2021;9:e11417.

Salas-Gismondi R, Flynn JJ, Baby P, Tejada-Lara JV, Wesselingh FP, Antoine PO. A Miocene hyperdiverse crocodylian community reveals peculiar trophic dynamics in proto-Amazonian mega-wetlands. Proc Biol Sci. 2015;282(1804):20142490.

Simpson GG. New reptiles from the Eocene of South America. Am Mus Novit. 1937;(927):1–3.

Skinner MF, Hibbard CW, Schultz CB. Early Miocene faunas from the Brule Formation, Nebraska. Bull Am Mus Nat Hist. 1967;132:1–35.

Speijer RP, Pälike H, Hollis CJ, Hooker JJ, Ogg JG. The Paleogene Period. In: Gradstein FM, Ogg JG, Schmitz ML, Ogg GM, editors. Geologic Time Scale 2020, Vol. 2. Amsterdam: Elsevier; 2020. p.1087–1140.

Stocker MR, Brochu CA, Kirk EC. A new caimanine alligatorid from the Middle Eocene of Southwest Texas and implications for spatial and temporal shifts in Paleogene crocodyliform diversity. PeerJ. 2021;9:e10665.

Tedford RH, Albright LB, Barnosky AD, Ferrusquia-Villafranca I, Hunt RM Jr, Storer JE, et al. Mammalian biochronology of the Arikareean through Hemphillian interval (late Oligocene through early Pliocene epochs). In: Woodburne MO, editor. Late Cretaceous and Cenozoic mammals of North America. New York: Columbia University Press; 2004. p.169–231.

Tchernov E, Van Couvering JAH. The Miocene crocodilian Brochuchus pigotti from Rusinga Island, Kenya. Israel J Earth Sci. 1978;27:209–220.

Walter J, Rabi M, Darlim G, Lee MSY. Fossil calibrations for molecular divergence dating of caimans (Crocodylia: Alligatoroidea: Caimaninae). Zool J Linn Soc. 2022;194(2):375–394.

Whiting ET, Head JJ. Paleobiogeography and systematics of Miocene alligatorids from Nebraska. J Vert Paleo. 2020;40(3):e1782411.

Willis RE, McAliley LR, Neeley ED, Densmore LD. Evidence for placing the false gharial (Tomistoma schlegelii) into the family Gavialidae: inferences from nuclear gene sequences. Mol Phylogenet Evol. 2007;43(2):787–794.

Wu XC, Russell AP, Brinkman DB. A review of Leidyosuchus canadensis Lambe, 1907 (Archosauria: Crocodylia) and an assessment of cranial variation based upon new material. Can J Earth Sci. 2001;38(12):1665–1687.
