# Supplementary material for: Competing methods of divergence time estimation recover diverse evolutionary timescales
Source: Proc Biol Sci. 2026 Oct 7;293(2080):20260635. doi: 10.1098/rspb.2026.0635 (PMC13641909; doi:10.1098/rspb.2026.0635)
Supplement: Supplementary file 1 [file rspb.2026.0635.zip › rspb.2026.0635/rspb20260635_si_001.zip!/supp/2.3_FigsS1-9.docx]

**Figure S1.** Node-calibrated prior timescale generated without sequence data and conditioned on a fixed topology. Time units are in millions of years (Ma). Blue bars indicate 95% HPD width for node ages.

**Figure S2.** Tip-calibrated prior timescale approximated without sequence data and conditioned on an extant backbone topology. Time units are in millions of years (Ma). Blue bars indicate 95% HPD width for node ages.

**Figure S3.** Tip-calibrated FBD prior timescale approximated without sequence data and conditioned on an extant backbone topology. Time units are in millions of years (Ma). Blue bars indicate 95% HPD width for node ages.

**Figure S4.** Node-calibrated posterior timescale generated with molecular data and conditioned on a fixed topology. Time units are in millions of years (Ma). Blue bars indicate 95% HPD width for node ages.

**Figure S5.** Node-calibrated posterior timescale generated with morphological data and conditioned on a fixed topology. Time units are in millions of years (Ma). Blue bars indicate 95% HPD width for node ages.

**Figure S6.** Node-calibrated posterior timescale generated with a combined dataset and conditioned on a fixed topology. Time units are in millions of years (Ma). Blue bars indicate 95% HPD width for node ages.

**Figure S7.** Tip-calibrated posterior timescale generated with a total-evidence dataset and conditioned on a fixed backbone topology. The phylogenetic position of fossil taxa was co-estimated and summarised using a 50% majority-rule consensus tree. Time units are in millions of years (Ma). Blue bars indicate 95% HPD width for node ages.

**Figure S8.** Tip-calibrated posterior timescale generated with a morphological dataset and conditioned on a fixed backbone topology. The phylogenetic position of fossil taxa was co-estimated and summarised using a 50% majority-rule consensus tree. Time units are in millions of years (Ma). Blue bars indicate 95% HPD width for node ages.

**Figure S9.** Tip-calibrated FBD posterior timescale generated with a total-evidence dataset and conditioned on a fixed backbone topology. The phylogenetic position of fossil taxa was co-estimated and summarised using a 50% majority-rule consensus tree. Time units are in millions of years (Ma). Blue bars indicate 95% HPD width for node ages.
